# X-linked muscular dystrophy in a Labrador Retriever strain: phenotypic and molecular characterisation

**DOI:** 10.1186/s13395-020-00239-0

**Published:** 2020-08-07

**Authors:** Inès Barthélémy, Nadège Calmels, Robert B. Weiss, Laurent Tiret, Adeline Vulin, Nicolas Wein, Cécile Peccate, Carole Drougard, Christophe Beroud, Nathalie Deburgrave, Jean-Laurent Thibaud, Catherine Escriou, Isabel Punzón, Luis Garcia, Jean-Claude Kaplan, Kevin M. Flanigan, France Leturcq, Stéphane Blot

**Affiliations:** 1 U955 - IMRB, Team 10 – Biology of the neuromuscular system, Inserm, UPEC, EFS, Ecole nationale vétérinaire d’Alfort, 94700 Maisons-Alfort, France; 2 Laboratoire de biochimie et génétique moléculaire, hôpital Cochin, AP-HP, université Paris Descartes-Sorbonne Paris Cité, Paris, France; 3 Laboratoire de Diagnostic Génétique-Institut de Génétique Médicale d’Alsace, Hôpitaux Universitaires de Strasbourg, 1 Place de L’Hôpital, 67091 Strasbourg, France; 4 Department of Human Genetics, The University of Utah School of Medicine, Salt Lake City, Utah USA; 5 SQY Therapeutics, Université de Versailles Saint-Quentin-en-Yvelines, Montigny le Bretonneux, France; 6 The Center for Gene Therapy, Nationwide Children’s Hospital, The Ohio State University, Columbus, Ohio USA; 7 Department of Pediatrics, The Ohio State University, Columbus, Ohio USA; 8 Sorbonne Universités, UPMC Université Paris 06, INSERM UMRS974, Centre de Recherche en Myologie, Institut de Myologie, G.H. Pitié Salpêtrière, Paris, France; 9 Aix Marseille Université, INSERM, MMG, Bioinformatics & Genetics, Marseille, France; 10 APHM, Hôpital Timone Enfants, Laboratoire de Génétique Moléculaire, Marseille, France; 11 Université de Versailles Saint-Quentin-en-Yvelines, U1179 INSERM, UFR des Sciences de la Santé, Montigny le Bretonneux, France

**Keywords:** Dog, Canine, Neuromuscular disorders, Animal model, DMD, LRMD, Dystrophin, Inversion, Dp71

## Abstract

**Background:**

Canine models of Duchenne muscular dystrophy (DMD) are a valuable tool to evaluate potential therapies because they faithfully reproduce the human disease. Several cases of dystrophinopathies have been described in canines, but the Golden Retriever muscular dystrophy (GRMD) model remains the most used in preclinical studies. Here, we report a new spontaneous dystrophinopathy in a Labrador Retriever strain, named Labrador Retriever muscular dystrophy (LRMD).

**Methods:**

A colony of LRMD dogs was established from spontaneous cases. Fourteen LRMD dogs were followed-up and compared to the GRMD standard using several functional tests. The disease causing mutation was studied by several molecular techniques and identified using RNA-sequencing.

**Results:**

The main clinical features of the GRMD disease were found in LRMD dogs; the functional tests provided data roughly overlapping with those measured in GRMD dogs, with similar inter-individual heterogeneity. The LRMD causal mutation was shown to be a 2.2-Mb inversion disrupting the *DMD* gene within intron 20 and involving the *TMEM47* gene. In skeletal muscle, the Dp71 isoform was ectopically expressed, probably as a consequence of the mutation. We found no evidence of polymorphism in either of the two described modifier genes *LTBP4* and *Jagged1.* No differences were found in *Pitpna* mRNA expression levels that would explain the inter-individual variability.

**Conclusions:**

This study provides a full comparative description of a new spontaneous canine model of dystrophinopathy, found to be phenotypically equivalent to the GRMD model. We report a novel large DNA mutation within the *DMD* gene and provide evidence that LRMD is a relevant model to pinpoint additional DMD modifier genes.

## Background

Duchenne muscular dystrophy (DMD) is an X-linked inherited disorder due to mutations in the Dystrophin gene. The large size of the gene explains the relatively high incidence of this genetic disease affecting around 1 in 5000 male live births [1]. Dystrophin-deficiency leads to severe muscle lesions including necrosis, regeneration, inflammation, fibrosis and fatty degeneration. Patients suffering from DMD initially present with gait difficulties and frequent falls and are usually diagnosed around 5 years of age [2]. Thereafter, the disease progresses rapidly and leads to a permanent use of the wheelchair at a mean age of 10 years without steroid therapy [3]. During adolescence and young adulthood, respiratory muscle weakness progresses leading patients to need ventilatory assistance, and a dilated cardiomyopathy develops [2]. Despite the significant progress in the medical management of these patients made over the last decades, respiratory and cardiac insufficiencies lead to premature death of these patients in the third to fourth decade of life (median 29.9 years in a recently published meta-analysis study) [4].

In parallel to this active clinical research that has substantially increased lifespan and quality of life of DMD patients, innovative therapies including gene, cell and pharmacological therapies are being developed with the aim of curing or alleviating DMD. Development of such strategies requires assessment of efficacy in animal models of the disease. Among the animal models of DMD, dystrophin-deficient dogs reproduce the main histopathological and clinical features of the human condition, in a large size organism [5, 6]. Therefore, they represent an excellent preclinical setting, compared to smaller animals, to ultimately validate the functional benefit of large-scale produced therapeutic agents, and better address translation to the clinical setting [6–8].

Twenty-nine spontaneous muscular dystrophy cases have been described in 26 different dog breeds, belonging to 9 of the 10 canine breed groups [6, 9–35]. Causative mutations in the dystrophin gene have been identified in 14 dog strains [6, 17, 23, 24, 28, 30–34, 36, 37] (listed in Table S2). In Golden Retrievers, a splice site mutation in intron 6 was found [38] and maintained in Golden Retriever background, or introgressed into Beagle background [39] rendering two models that have been extensively used in preclinical studies for DMD.

The Golden Retriever muscular dystrophy (GRMD) dog model is well known and has been thoroughly characterised; clinical signs include early and progressive gait impairment, dyspnoea, dysphagia and delayed dilated cardiomyopathy, altogether resembling the human DMD clinical profile. The model also displays the strong inter-individual heterogeneity found in humans, currently an active field of investigation to identify disease modulators [5, 40–42]. Quantitative evaluation tools, often adapted from techniques used in patients, have been developed for the GRMD dog model to support the use of this model in preclinical efficacy studies [43–46]. However, this model is sometimes criticised because of the inter-individual heterogeneity and its low availability [7, 47]. The GRMD mutation is located out of the DMD major hotspot of mutation and is therefore not a perfectly similar genetic context for most of DMD cases [24]. In addition, the presence of revertant fibres expressing a large part of the full-length dystrophin might be confusing by conferring immune tolerance to engineered dystrophins [8]. Therefore, the interest of new canine models, alternative or complementary to GRMD, should be investigated each time a spontaneous case is identified. Recently, an intron 50 mutation identified in Cavalier King Charles, introgressed and maintained in a beagle background, has been used to provide proof of concept of successful targeted gene editing in DMD dogs [48].

Here, we report a canine X-linked muscular dystrophy in a Labrador Retriever strain, named Labrador Retriever muscular dystrophy (LRMD), for which a breeding colony was established, in order to evaluate the possible interest of this model in genetic or preclinical efficacy studies. In this paper, we provide a comprehensive molecular and clinical characterisation as well as functional assessment of the LRMD model and compare it to the well-established GRMD dog model.

## Methods

### Animals and colony establishment

The two first affected dogs (LRMD1 and 2) belonged to a Labrador Retriever breeder, and were referred to the neurology consultation of the Alfort school of veterinary medicine (EnvA) at the age of 4 months by their veterinarian. These animals underwent a complete neurological assessment, measurement of serum CK activity, electrodiagnostic studies and muscle biopsies. Subsequently, the breeder donated two affected brothers from another litter (LRMD3 and 4) as well as a sister with a 50% risk to be a carrier to the Neurobiology laboratory in EnvA. A first litter born from this female and the LRMD4 male yielded an affected male and an affected female attesting the heterozygous status of the dam. A colony was then established and maintained using crossings between affected males and carrier females. The reproduction was assisted by artificial insemination. A total of 10 litters rendered 26 LRMD dogs among which 10 survived beyond 1 month of age. A total of 14 LRMD dogs (9 males and 5 females) were studied (4 born in the breeding farm and 10 in the EnvA facilities). Prior to availability of a genetic testing, LRMD dogs were diagnosed based on serum CK values and on clinical and histological observations; the unaffected females were *de facto* considered as heterozygous due to the crossing pattern used (diseased father). For ethical reasons, the breeding of this colony has now been stopped and frozen semen from LRMD males has been stored in order to be able to rebuild a colony if needed.

### Survival analysis

A Kaplan-Meier survival curve was performed including all the LRMD (*n* = 30). Given the fact that the GRMD dogs were not bred in the laboratory but at an external breeding farm and arrived in the lab when they were 2-month-old, the comparison of the survival between both colonies was performed from the age of 2 months. The 14 LRMD dogs that survived to 2 months of age were compared with 181 GRMD dogs that were not included in any systemic treatment protocol, and to 14 healthy dogs from the GRMD colony. Survival data were censored in two cases: if a dog was still alive at the time of the analysis, or if the dog left the laboratory to be adopted in the case of healthy dogs. Percentiles of survival were calculated using the software Statistica 10 (StatSoft ®). Survival data between GRMD and LRMD was compared using a log-rank test.

### Clinical scoring

Dogs were clinically scored using the grid developed and validated in the laboratory for GRMD dogs [49]. Seven LRMD dogs (three females, four males) underwent iterative clinical scoring from weaning (2 months of age) and throughout their life. The clinical scores were compared to the values obtained from an age-matched male GRMD population encompassing 21 animals, and statistical significance was assessed by means of *t* tests. The dispersion of the data obtained for each population was evaluated by calculating the coefficients of variation. Seven LRMD dogs (three females, four males) underwent clinical scoring at an adult age (mean 23.6 months, SD 12.0 months); animals scored at adulthood included 5 out of the 7 animals scored from 2 months of age. The data were compared to the data obtained from eleven adult male GRMD dogs (mean 23.2 months, SD 13.9); there was no statistically significant difference in age among the two groups (*p* = 0.9).

### Gait analysis using 3D-accelerometry

The gait quality was quantified using a method previously described in GRMD dogs based on 3D-accelerometric recordings near to the centre of gravity during spontaneous locomotion [50].

Three LRMD dogs (two females, one male) were tested iteratively during the progressive phase of the disease. The first test was performed when the animals were between 2 and 3 months of age, and the dogs were subsequently tested every 2 weeks until the age of 9 months. The results were compared to those of a population of 24 age-matched male GRMD dogs and 6 healthy male Golden Retrievers. Four LRMD dogs (2 females, 2 males) were tested at an adult age (mean 20.7 months, SD 10.4 months). As previously described [51], a principal component analysis (PCA) was performed using 7 variables (stride frequency, stride length, regularity, three relative powers and total power); 18 male GRMD (mean age 17.8 months, SD 14.5 months) and 11 healthy male adult Golden Retriever dogs (mean age 21.3 months, SD 21.9 months) were used as active individuals. This reference PCA plane represents 90.6% of the variance (68.2% by component 1 and 22.4% by component 2). In order to position the LRMD dogs relative to GRMD and healthy dogs, they were projected as supplementary individuals on this PCA plane.

### Force measurement

An in vivo force measurement on both pelvic limbs was performed at the age of 4 and 6 months in two LRMD dogs (one male, one female), as previously described [52]. The maximal tetanic isometric force was obtained by averaging the values of six tetanic contractions obtained at 50 Hz and using a supramaximal stimulation of the fibular nerve. For the analysis, and to compare animals with different size (healthy dogs are larger than diseased ones), the absolute force value was normalised by the length of the leg measured in the force measurement device, leading to a relative force index (N/m). Since an incomplete relaxation after a tetanic contraction is frequently seen in GRMD dogs [52], this type of abnormality was also assessed in LRMD dogs. The post-tetanic residual contraction index was calculated as the difference between the baseline resting values before and after the tetanic contraction and expressed as a percentage of the tetanic absolute force. The results obtained in the LRMD dogs were compared to age-matched values obtained from 8 healthy and 17 GRMD age-matched dogs, examined at 4 and 6 months of age. Given the low number of LRMD dogs tested, this comparison remained qualitative (no statistical test performed).

### Respiratory function analysis

Two different methods were used to evaluate respiratory function in LRMD dogs [53]. The first method used is based on fluoroscopic acquisitions focused on the diaphragmatic region performed using a fluoroscopy imaging system (GE® OEC 9800). Conscious dogs were positioned in right lateral recumbency, reassured and gently maintained in this position for a period of 9 s to perform the acquisition at 25 Hz. End-inspiratory and end-expiratory images were used to calculate the range of motion of the diaphragm. The ventral edge of the caudal vena cava foramen was tagged in both images that were thereafter superimposed in order to measure the distance between both tags using Photoshop CS3 software. The diaphragm excursion distance was normalised by the length of the 13th thoracic vertebra captured on the images in order to account for differences in size between dogs. A second index was calculated on end-expiratory images, to study the resting position of the diaphragm. The angle formed at the ventral edge of the diaphragmatic foramen of the caudal vena cava, between a line perpendicular to the vertebral axis and a line joining the caudal edge of the 11th thoracic vertebra was measured using the Visilog 7.0 software. The smaller the angle is, the more caudally retracted is the diaphragm. Four adult LRMD dogs (two males and two females) were tested at a mean age of 24.0 months (SD 17.1 months), and compared with five adult healthy male dogs (mean 15.2 months of age SD 5.3 months) and 11 adult GRMD male dogs (mean 25.0 months, SD 24.3 months).

The second method used was the analysis of tidal breathing flow-volume loops, acquired on conscious dogs. A pneumotachometer (Spirobank II, MIR®) was linked to a tight-fitting facemask and placed over the muzzle of the animal including the lip commissures. Tidal breathing flow-volume loops were acquired and analysed using WinSpiro PRO (MIR®) software. Loops without artefacts were selected and the following measurements were performed: peak expiratory flow (PEF), peak inspiratory flow (PIF) and expiratory flow once 75% of the expiratory volume was reached (EF75). Two ratios were then calculated: the PIF/PEF and the EF75/PEF [53]. Four adult LRMD dogs (two males and two females) were tested at a mean age of 24.9 months (SD 16.0 months), and compared with eight adult healthy male dogs (mean 23.1 months of age, SD 8.1 months) and 11 adult GRMD male dogs (mean 25.7 months, SD 25.0 months).

Statistical significance between LRMD and healthy or GRMD dogs was assessed using *t* tests.

### Echocardiography

Two LRMD dogs were examined using echocardiography: LRMD4 at 5 months of age, and LRMD7 at 5.4 and 7.4 years of age. Dogs were not sedated during the imaging session. Conventional 2D and M mode echocardiography was performed. In particular, a right parasternal short axis view 2D-guided M-mode acquisition was used to calculate the shortening fraction (%). In addition, tissue Doppler imaging was performed in the youngest dog and the difference between systolic left free-ventricular endocardial and epicardial velocities was calculated and named myocardial gradient of velocities, as previously described [54].

### Histological techniques

Muscle biopsies were collected either during necropsy or during a surgical procedure, using the anaesthesia protocol used for the muscle force measurement with morphine as per-operative analgesia. They were immediately snap-frozen in isopentane cooled in liquid nitrogen and kept at − 80°C until sectioning. Sections of 10 μm were used for morphological staining, and sections of 7 μm were used for immunohistological experiments.

#### Histopathological evaluation using H&E staining

Dried sections were stained 10 min in Hematein and 5 min in 1% Eosin, dehydrated in four consecutive baths of ethanol and mounted in Canada balsam after soaking in xylene. A total of 112 biopsies from different skeletal muscles of diseased dogs of ages ranging from 0 to 76 months were sampled and qualitatively evaluated. Thirty-eight of these biopsies were collected during the necropsy of neonate animals. Thirty-three of the 112 samples obtained from different skeletal muscles (*biceps femoris*, s*artorius cranialis*, *tibialis cranialis*, *vastus lateralis*, *triceps brachii*, *extensor carpi radialis* and diaphragm), collected at different ages ranging from 2 to 24 months were analysed using a pathological quantification method based on a random sampling and annotation of histological events on entire sections as previously described [52].

#### Alizarin red S staining

Dried sections were stained during 5 min in a 2 % alizarin red S solution at pH 5.4. Thereafter, samples were rinsed and dehydrated in acetone and acetone-xylene volume/volume, and mounted in Canada balsam. Fibres with a normal calcium content appeared pale pink, whereas calcium overloaded fibres acquired a deeper shade ranging from deep pink, to bright red for the more strongly overloaded. This staining was used on the 38 biopsies from deceased neonates in order to investigate a potential neonatal form.

### Immunohistochemistry

Seven-micrometers sections from each biopsy were dried, rehydrated and fixed in cold acetone-methanol. Thereafter, samples were incubated with a 1:20 dilution of one of the following anti-dystrophin antibodies: MANEX1A (*N*-terminal part of dystrophin), MANEX1011C (rod domain repeat 1), MANDYS107 (rod domain repeat 15), kindly gifted by Pr Glenn E. Morris (CIND) and Novocastra NCL-Dys2 (C-terminal part) and NCL-Dys1 (rod domain repeats 8-9). Secondary antibodies used were anti-mouse FITC (1:50) or Cy3 (1:800) (Jackson Immunoresearch Laboratories Inc.).

### Western blot

#### Multiplex Western blot

Muscle protein analysis, including dystrophin, was performed using muscle surgical biopsy samples by multiplex Western blot analysis using the procedure described by Anderson and Davison [55] . Monoclonal antibodies used for multiplex Western blots were from Novocastra (Newcastle, United Kingdom; www.novocastra.co.uk). Multiplex Western blots combinations of antibodies used were as follows: (1) multiplex A includes antibodies Dys8/6C5 (NCL-DYS2/dystrophin C-ter), Cal3c/2A2 (NCL-CALP-12A2/calpain 3 exon 8), 35DAG/21B5 (NCL-g-SARC/g-sarcoglycan) and Ham1/7B6 (NCL-Hamlet/dysferlin); (2) multiplex B includes the antibodies Dys4/6D3 (NCL-DYS1/dystrophin rod domain), Calp3d/2C4 (NCL-CALP-2C4/calpain 3 exon 1) and ad1/20A6 (NCL-a-SARC/a-sarcoglycan).

#### Dystrophin Western blot

Total protein was extracted from muscle sections by treating the samples with 250 mM sucrose, 10 mM Tris pH 7.6, 0.1 mg/ml leupeptin, 1 mg/ml aprotinin and 20 mg/ml PMSF. After protein quantification (BCA kit, Pierce), proteins were denatured by incubating the samples in a solution containing 20% SDS, 20% sol, 20% glycerol, 10% β-mercaptoethanol, and 12.5% migration buffer at 100 °C during 3 min. Then, 250 μg proteins from two muscles biopsies from LRMD 3 (*biceps femoris*, *interosseous*) and 50 μg of total protein obtained from the *biceps femoris* of a healthy dog were loaded and migrated on a 4–12% NuPAGE Bis-Tris polyacrylamide gel (Life Technologies ®). Proteins were subsequently transferred onto a nitrocellulose membrane, the immunoblotting was performed using NCL-Dys2 antibody (Novocastra, 1:50) and secondary horseradish peroxidase-conjugated antibody (1:1000). The membrane was visualised using ECL (Amersham).

### Identification of the mutation

#### RT-PCR screening of the Dp427m transcript

Total RNA was extracted from muscle biopsies from LRMD3 and a healthy littermate using the RNeasy kit (QIAGEN®). Reverse transcription was performed using the Superscript II RT kit (Invitrogen®). The Dp427m transcript was explored using nested RT-PCRs with nine pairs of primers designed to amplify overlapping segments of the cDNA, based on the published canine dystrophin cDNA sequence (ESNCAFT00000036277) as follows: exons 3 to 10, exons 10 to 20, exons 15 to 22, exons 21 to 26, exons 25 to 36, exons 35 to 46, exons 45 to 56, exons 55 to 67 and exons 66 to 79 (sequences in Table S1). For these RT-PCR experiments, the PCR MasterMix kit (Promega) was used.

#### Southern Blot analysis

Genomic DNA from two LRMD dogs (LRMD3 and LRMD4) as well as from two unrelated healthy Labrador retriever dogs was studied by Southern blotting following EcoRI restriction. After electrophoresis on 0.8% agarose gel, the DNA was transferred from the gel to a nitrocellulose membrane by passive diffusion. Blots were hybridized with three dystrophin ^32^P-labeled cDNA probes: probe 1 encompassing exons 18 to 24, probe 2 covering exon 21 and probe 3 covering exon 20. After washing, the membrane was exposed on an autoradiography film at − 70 °C.

#### PCR of intron 20

Genomic DNA was extracted from blood using DNeasy kit (QIAGEN). Four pairs of primers named F1-R1 to F4-R4 were designed to amplify the complete intron 20 in four overlapping amplicons using the published canine dystrophin gene sequence (ENSCAFG00000023562). F1 was designed to bind to exon 20, and R4 to exon 21 (sequences in Table S1). Supplementary primers (F4i, and R4i) were designed to amplify a region between F4 and R4 in order to better explore this zone. Sanger sequencing of the PCR products was performed after DNA gel extraction using the QIAquick gel extraction kit (QIAGEN).

#### RNA-seq analysis

Frozen muscle biopsies were homogenized in 20 mM Tris HCl pH 7.4, 150 mM NaCl, 5 mM MgCl_2_, 1 mM DTT and 1% Triton X-100, and total RNA was extracted from using TRIzol® LS reagent (Life Technologies) followed by isopropanol precipitation according to the manufacturer’s instructions. Cytoplasmic and mitochondrial rRNA was depleted from ~ 1 μg of total RNA using tiling oligodeoxynucleotides and the RNase H digestion method [56]. Illumina TruSeq Stranded Total RNA library kits were used to prepare random-primed, indexed RNA-seq libraries from the rRNA-depleted total RNA preparations and these libraries were sequenced on an Illumina HiSeq 2500 instrument using single-end 50 bp v4 read chemistry. Quality metrics for FASTQ reads were evaluated with *FastQC* tool (http://www.bioinformatics.babraham.ac.uk/projects/fastqc) and reads were quality trimmed and adapter clipped using the *Trimmomatic* tool [57] with standard parameters. FASTQ sequence reads were mapped to the Broad CanFam3.1/canFam3 (Sep. 2011) reference genome using the *STAR* v2.7 RNA-seq aligner [58]. Strand-specific coverage plots were generated from the alignment files using the BEDTools genomecov function and displayed in the UCSC genome browser after conversion with the UCSC bedGraphToBigWig utility.

#### Genetic diagnostic test development

In order to reliably identify healthy, carrier and affected animals, a PCR-based genetic test was developed. The F4-R4 pair of primers corresponding to the 5′ end of intron 20 was used to amplify the WT allele. A pair of primers were designed to amplify across the distant breakpoint, named mutF-mutR (normal size 1939 bp) and supposed to amplify the WT allele but not the LRMD. PCR was set up using the Phusion high fidelity DNA polymerase (Thermo Scientific ®). The LRMD F4-mutR and mutF-R4 bands were extracted from the gel using a Nucleospin Gel and PCR Cleanup kit (Macherey-Nagel), and Sanger sequencing was performed in order to characterise the mutation breakpoints. The diagnostic PCR was designed to be run with three primers in a single PCR: F4, R4 (1661 bp band WT allele) and mutR (890 bp band LRMD allele).

### RT-PCR of the Dp71 transcript

Total RNA was extracted from tissues using the RNeasy kit (QIAGEN ®). Samples from the following tissues were used: liver from a healthy dog (Dp71 expression positive control), *biceps femoris* muscle from a healthy dog, a GRMD dog and a LRMD dog (LRMD8) and *sartorius cranialis*, *tibialis cranialis* muscles from a LRMD dog (LRMD8). Reverse transcription was performed using the Superscript II RT kit (Invitrogen®). cDNA concentration was determined by spectrophotometry (Nanodrop). PCR was performed on 120 ng of each of the 7 cDNA samples using the Phusion high fidelity DNA polymerase (Thermo Scientific ®). The forward primer was designed to bind to the junction of the first specific exon of the Dp71 transcript and the exon 63 of the Dp427 transcript (exon 2 of the Dp71), the reverse primer was designed to bind to the junction between exons 64 and 65 of the Dp427 transcript (exons 3–4 of the Dp71) based on published canine sequences AY566609 and ENSCAFT00000036277 (Sequences in Table S1). The expected size of the PCR product was 164 bp.

### Jagged1 PCR and sequencing

A PCR involving the G>T point mutation described to be associated with very mild clinical forms in GRMD dogs was run in 5 LRMD dogs with diverse phenotypes, using the primers published in [41] and the Phusion high fidelity DNA polymerase (Thermo Scientific ®). Sanger sequencing of the PCR products was then performed in order to assess the presence of this point mutation in the promoter region of the *Jagged1* gene.

### Pitpna mRNA expression

*Pitpna* mRNA expression was compared between two severely affected LRMD dogs (LRMD8 and 9, loss of ambulation/poor mobility, respectively), and two less severely affected LRMD dogs (LRMD7, the oldest survivor, and LRMD14, the mildest locomotor form). RNA was obtained from frozen biopsies of *biceps femoris* sampled at the age of 2 months using the NucleoSpin RNA Plus kit (Macherey&Nagel). Further, 300 ng of total RNA were retro transcribed with the Maxima First Strand cDNA Synthesis Kit (Thermofisher). The cDNA obtained was run in triplicates and qPCR Maxima SYBR Green (Thermofisher) used as reagent for real time PCR in a Light Cycler 96 (Roche). The amplification protocol used is as follows: pre-incubation at 95 °C for 5 min, followed by 45 cycles at 95 °C for 15 s, 59 °C for 20 s and 72 °C for 20 s. At the end, a cycle of high-resolution melting was added. The DDCt method was used to determine the expression level of *Pitpna* gene versus *ß-Actin* (housekeeping gene). The sequences of the primers used are described in [42]. A repeated-measures ANOVA was used to assess statistical significance.

### LTBP4 SNPs analysis

The Ensembl genome browser was used to identify known polymorphisms in the 30 coding exons of the canine latent transforming growth factor beta binding protein 4 (*LTBP4*) gene. Genotypes at these variant sites were called from the RNA-seq data from 5 LRMD dogs (LRMD 8, 9, 10, 12 and 13) using the Genome Analysis Toolkit (GATK, https://software.broadinstitute.org/gatk) best practices workflow for SNP and indel calling on RNA-seq data. The known sites in *LTBP4* ENSCAFG00000005133 included 4 missense SNP variants (Ensembl Variant ID: rs851496729:p.Pro425Thr, rs851016279:p.Ser439Pro, rs853156464:p.Pro545Ala and rs21954920:pAla668Thr) and 7 synonymous SNP variants (Ensembl Variant ID: rs851354589, rs21999801, rs21954918, rs853084197, rs851625070, rs852260326 and rs850783330); no novel coding variants were observed from the LRMD RNA-seq data.

For all the statistical tests performed, the level of statistical significance was set at *p* ≤ 0.05.

## Results

### Report of the first cases

**Fig. 1:**
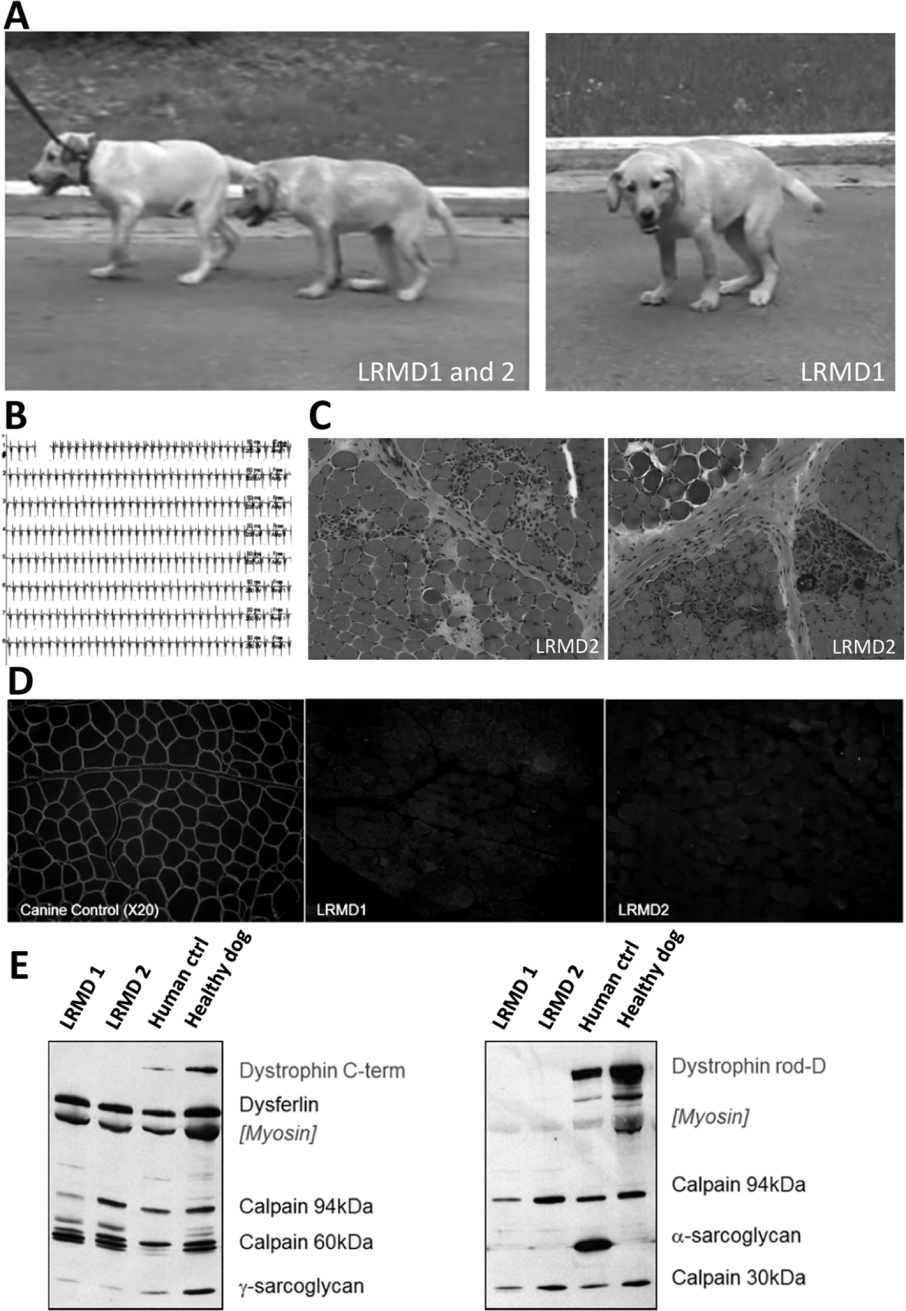
Findings it the first cases point out a potential dystrophinopathy. **a** Pictures of the two first affected brothers (LRMD1 and 2), at 4 months of age, at time of their presentation to the neurology consultation of the Alfort school of veterinary medicine. Note the markedly plantigrade and palmigrade posture and the pelvic verticalisation. **b** Electromyographic recordings showing complex repetitive discharges observed in both animals, especially in the proximal appendicular muscles. **c** H&E staining of a *biceps femoris* biopsy taken from LRMD2 at 4 months of age showing a dystrophic profile: necrosis, regeneration, endomysial fibrosis, hypercontracted fibres, inflammatory foci and calcifications. **d** Dystrophin (rod-domain) immunostaining in LRMD1 and 2 *biceps femoris* compared to an unaffected dog. The images clearly show absence of signal delineating the membrane of the muscle fibres in LRMD dogs and presence of this signal in the images corresponding to the control dog. **e** Multiplex Western-blot confirming the absence of dystrophin immunoreactivity in both affected dogs using two different anti-dystrophin antibodies (Dys2, anti-C-terminal part, and Dys1, anti-rod domain). γ-Sarcoglycan was present in both affected dogs but levels were lower than in the healthy dog, probably because of disruption of the dystrophin-associated protein complex. The levels of other studied proteins were unmodified except for the α-sarcoglycan, for which the human antibody showed no cross-reactivity in canine muscles

Two 4-month-old male Labrador Retriever littermates owned by a breeder were referred by their veterinarian to the neurology consultation of the Alfort school of veterinary medicine. The puppies presented with gait stiffness, exercise reluctance and marked palmigrade and plantigrade posture (Fig. 1a). At the clinical examination, normoreflexia and absence of proprioceptive defect were observed. Both puppies had markedly elevated serum creatine kinase (LRMD1: 83,000 UI/L; LRMD2: 30,000 UI/L), and the electrodiagnostic studies revealed normal nerve conduction velocities, but spontaneous activity was evidenced in all the tested muscles (mainly complex repetitive discharges, Fig. 1b). Biopsies from the *biceps femoris* muscle were sampled from both dogs and histological analysis revealed lesions of active necrosis, regeneration, endomysial fibrosis and a few inflammatory foci with calcifications (Fig. 1c). The phenotype of these two dogs, together with the high CK, the myopathic electrodiagnostic profile and the histological lesions were suggestive of a muscular dystrophy process. Immunostaining of the biopsies showed no reactivity with an anti-dystrophin antibody directed against the central rod domain (Dys1, Fig. 1d). Analysis of muscle tissue by multiplex Western blot (Fig. 1e) confirmed the absence of Dp427 dystrophin and the presence of normal size dysferlin, γ-sarcroglycans and calpain 3; proteins altered in human limb-girdle muscular dystrophies. A new litter born from the same parents yielded two additional affected males, reinforcing the hypothesis of an X-linked transmission. The breeder kindly gifted these two affected males (LRMD3 and 4) as well as an unaffected female born from the same parents to our research unit. This allowed us to establish a LRMD colony in the same facilities in which a pre-existing GRMD colony had already been established.

### LRMD colony establishment

A first litter was obtained by crossing the presumed female carrier and one of the affected males (LRMD4). The subsequent litters were obtained by crossing the descendant dogs (carrier females×affected males). The inbreeding of the colony was high (ranging from 25% for LRMD5 and 6, to 41% for LRMD11 to 14). Over a period of 6 years, ten litters were obtained (Figure S1). A total of 14 LRMD dogs (9 males and 5 females) survived the neonatal period and were followed-up.

### Main phenotypic features

#### Neonatal period

At birth, the LRMD dogs had weights that were comparable to that of their healthy littermates. Shortly after birth, these animals showed striking difficulties sucking and needed intensive nursing during their first days to ensure survival (feeding by oro-gastric gavage and keeping in incubator when required). Despite this care, around 50% of the LRMD newborn puppies died within the first 48 h (Figure S2) following a paroxystic weakness episode associated with severe dyspnoea. Myoglobinuria was observed in some of these puppies, as well as very high serum CK values (> 100,000 UI/L) and hyperkalaemia. Rhabdomyolysis was confirmed by histological analysis. Selective involvement of some muscles such as the diaphragm, the tongue or the *sartorius cranialis* (Figure S2) was observed, confirming that this neonatal syndrome reproduces the neonatal fulminating form well described in the GRMD dog model [59].

#### Clinical observations

##### Locomotor signs

After few days of intensive nursing, the surviving LRMD puppies became able to suck on their own but exhibited growth retardation compared to their healthy littermates (Figure S2). At the age of 2 months, the LRMD puppies showed stiff gait and difficulty jumping over an obstacle. In the subsequent months, they became stiffer, and rapidly developed posture abnormalities resembling those seen in GRMD dogs notably marked pelvic verticalisation, palmigrady and plantigrady. When the LRMD dogs reached 6 months of age, most of them were unable to run and showed abnormal gait while walking. Despite the abnormalities, most animals remained ambulant, except for LRMD8, who completely lost ambulation at 6 months of age and was therefore euthanized. His littermate (LRMD9) also developed a severely compromised locomotion.

##### Respiratory and digestive signs

All the LRMD dogs developed signs of dyspnoea at the age of 2–3 months, mainly paradoxical thoraco-abdominal movements. In the following months, the dogs developed moderate exercise-induced polypnoea, noisy breathing and in some cases intermittent cyanosis and elevated serum bicarbonate concentration. Oro-pharyngeal dysphagia became a major feature from the age of 4 months onwards, with a prominent aggravation in the following months. This severe dysphagia probably resulted from markedly reduced jaw opening, together with prominent macroglossia, retraction of the tongue and weakness of the pharyngeal muscles. The symptoms were observed in all LRMD dogs, and in some cases, they limited their ability to maintain their water intake; consequently, the deterioration of 50% of the animals was cause for humane euthanasia. The oro-pharyngeal dysphagia observed in the LRMD dogs was frequently complicated with aspiration bronchopneumonias that were successfully treated in most cases, but led to the death of 4 out of the 14 affected dogs. The chest radiographs showed usual abnormalities found in GRMD dogs: hiatal hernias, megaoesophagus, *pectus excavatum*, pulmonary hyperinflation associated with diaphragm flattening [60].

##### Survival

**Fig. 2:**
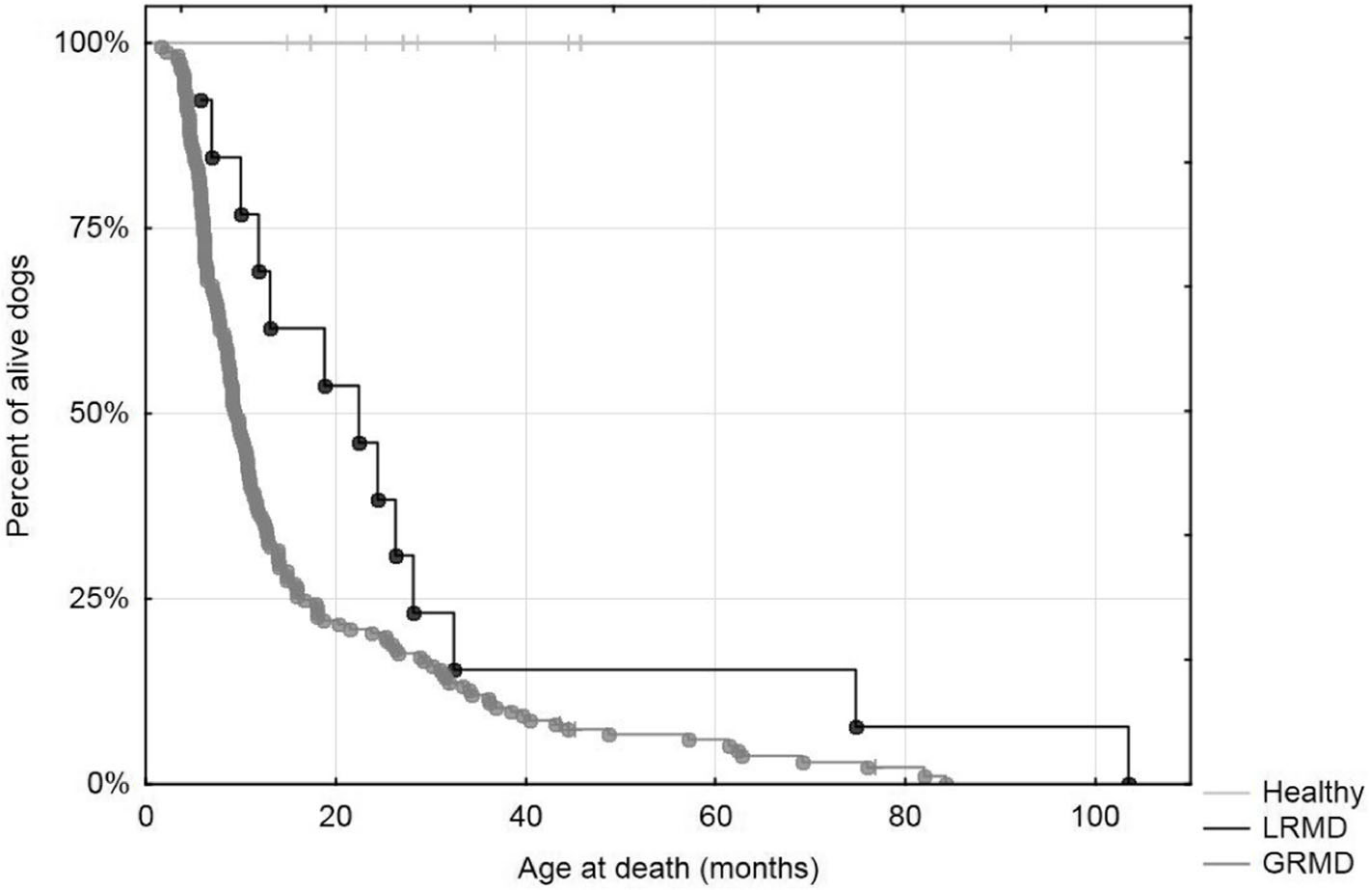
Survival of LRMD dogs. Kaplan-Meier survival curve comparing LRMD, GRMD and healthy dogs excluding the neonatal period (beginning of the follow-up at 2 months of age). Survival was markedly reduced in both models compared with healthy dogs. LRMD dogs had a slightly better survival rate and this trend was statistically significant in the log rank test (*p* = 0.01)

The mean survival of these 14 LRMD dogs was 21.6 months, with a first quartile at 10.8 months and a third quartile at 31.4 months. The LRMD dog with the longest survival (LRMD7) died at the age of 103.5 months (8.6 years), probably following paroxystic cardiac arrhythmias (sudden death without significant findings in the necropsy in a dog known to have prominent ventricular arrhythmias). Despite this long survival, this dog exhibited impaired ambulation, dyspnoea, dysphagia and suffered many aspiration pneumonia episodes. Consequently, this animal cannot be considered a ‘phenotypic escaper’. A comparative survival analysis was performed between LRMD and GRMD dogs and showed that LRMD dogs tended to live longer (log-rank test *p* = 0.01) (Fig. 2).

#### Histological observations

The studied biopsies showed general trend towards necrosis-regeneration lesions in LRMD dogs younger than 1 year. Inflammatory foci and calcifications were observed in some biopsies from 4- and 6-month-old dogs. As the animals grew older, endomysial fibrosis became prominent and most animals beyond 2 years of age suffered adiposis (Figure S3). In parallel, the CK values, although fluctuating, were markedly elevated during the first year, and decreased to lower values in older LRMD dogs. The most severely affected muscles were the diaphragm and the *extensor carpi radialis* with pathological indices > 60%.

#### Functional evaluation and comparison with the GRMD model

The overall phenotypic characteristics of the LRMD dogs resembled those observed in GRMD dogs. In order to position the model in comparison to the ‘reference’ GRMD dog model, a quantitative comparative study between both canine muscular dystrophies was performed, using the tools developed to evaluate GRMD dogs.

##### Clinical score

**Fig. 3:**
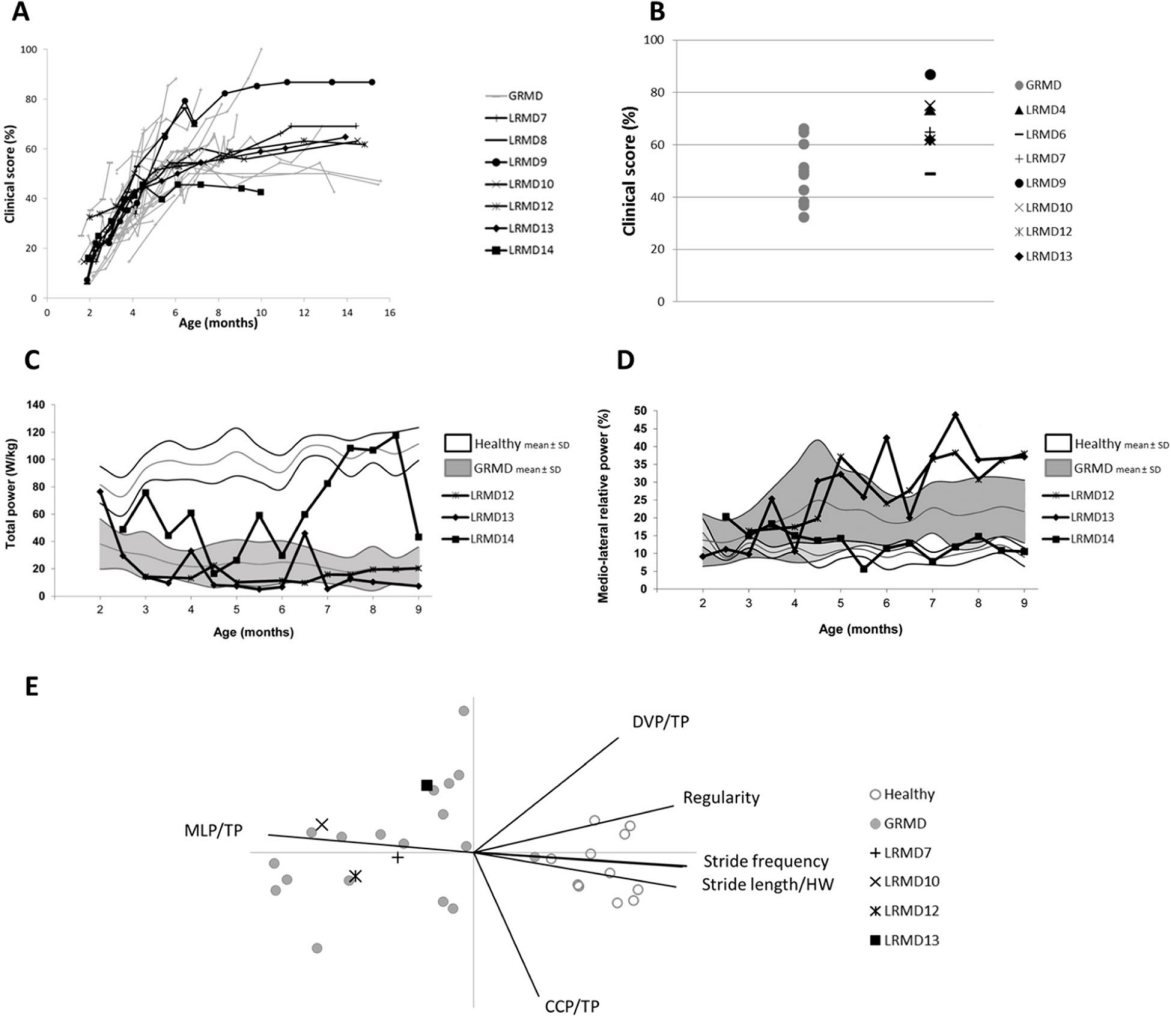
Clinical scoring and gait analysis in LRMD dogs. GRMD dogs are represented in grey, and LRMD dogs in black. **a** longitudinal evolution of the clinical score during disease progression. The clinical score was low at the age of two months in both colonies, thereafter it rapidly increased to roughly stabilise after the age of 8 months; this indicates that there is rapid disease progression during the first months of life. From 2 to 4 months, all animals showed rapid worsening of clinical signs, with a tendency towards more homogeneous evolution of LRMD dogs. From 4 months of age onwards, the clinical evolution of the animals became more heterogeneous, with two dogs presenting more severe phenotype, and one animal presenting milder phenotype. At later stages, the clinical scores of LRMD dogs were higher than those of GRMD dogs. This was confirmed by comparing adult dogs. **b** comparison of clinical scores in adult LRMD vs. GRMD dogs. The clinical scores obtained in adult, clinically stabilised LRMD dogs were significantly higher than those observed in GRMD dogs (*p* = 0.005), suggesting that adult LRMD dogs were globally more affected than GRMD dogs. **c**, **d** longitudinal follow-up of 3 LRMD dogs during the progressive phase of the disease. The white and grey areas, respectively, represent the mean ± 1 SD of healthy dogs and GRMD dogs; the black curves represent the individual evolution of each of the three LRMD dogs. **c** Evolution of total power from the age of 2 to the age of 9 months. Two among the three LRMD dogs had markedly decreased total power values, the levels observed were comparable to those observed in GRMD dogs. The third animal (LRMD14) exhibited rather preserved total power values, especially in the second part of the follow-up, from the age of 6.5 months onwards, where some values overlapped with those of healthy dogs. **d** Evolution of the relative medio-lateral power from the age of 2 to the age of 9 months. Two among the three LRMD dogs showed a dramatic increase of their medio-lateral relative power with age and disease progression, while the third dog (LRMD14) maintained normal values throughout the follow-up period. **e** Projection of 4 adult LRMD dogs as supplementary individuals on a PCA (principal component analysis) plane built with adult healthy and GRMD dogs as active individuals. Healthy and GRMD populations are well separated and LRMD dogs appear in the cloud of GRMD dogs. The straight lines represent the projection of variables. The dystrophic population was separated from the healthy population mainly by decreased total power, stride length and frequency, and increased medio-lateral power. *MLP*/*TP* medio-lateral power/total power, *DVP*/*TP* dorso-ventral power/total power, *CCP*/*TP* cranio-caudal power/total power, *HW* height at withers

Clinical scoring rapidly increased during the first months in LRMD dogs; thereafter, the score progressed at slower rate until the animals reached the age 7–8 months when it stabilised. The same type of evolution was seen in the GRMD population (Fig. 3a). However, when comparing both colonies in detail, the LRMD dogs had a faster and more homogeneous disease progression within the first months. At the age of 4 months, the LRMD dogs tended to have higher scores (*p* = 0.07) and the coefficient of variation of the clinical score was lower than that of the GRMD colony (12% vs. 34% in GRMD dogs). Thereafter, two subpopulations of LRMD dogs emerged, the first one formed by a group of two animals that were more severely affected from a locomotor point of view (LRMD8 and 9). This suggests that, as previously described for the GRMD model, there may be a severe form leading to a loss of ambulation in LRMD dogs [40]. The less severely affected LRMD dogs tended to have higher clinical scores than GRMD dogs between the ages of 10 and 16 months. These observations were confirmed by comparing the colonies at an adult age (Fig. 3b); stage at which LRMD dogs had significantly higher clinical scores (mean 67.5%, SD 12.1%) than GRMD dogs (mean 48.1%, SD 11.7%; *p* = 0.005). It is noteworthy that LRMD7, the oldest survivor, had clinical scores among those measured in other LRMD dogs.

##### Gait analysis

Gait analysis of LRMD dogs using accelerometry indicated that both the LRMD and the GRMD models exhibited similar profiles compared to healthy dogs: decreased speed, stride length and frequency, decreased total power and increased medio-lateral relative power. Among the three LRMD dogs longitudinally followed-up (Fig. 3c, d), two showed values that were similar to those of more affected GRMD dogs, as well as a decrease in gait quality with age and dramatic increase in gait waddle. The third dog (LRMD14) had mild gait impairment, attesting to the existence of inter-individual heterogeneity in LRMD dogs. Despite this heterogeneity and the low number of animals followed-up, the total power, a very discriminating variable, was found to be significantly decreased at all tested ages (*p* = 0.035 at 4 months of age, *p* = 0.0001 at 6 months of age, *p* = 0.002 at 9 months of age), similarly to GRMD dogs (no significant difference between both models). The four LRMD dogs examined at an adult age obtained gait index values overlapping with those usually measured in the GRMD model and significantly different from healthy dogs (*p* = 0.036 for the stride frequency to *p* < 0.0001 for the total power). No significant difference was found between LRMD and GRMD dogs, though the relative medio-lateral power tended to be higher in LRMD dogs (*p* = 0.059, mean LRMD = 42% (SD = 10%), mean GRMD = 29% (SD = 13%)). When projecting these four dogs as supplementary individuals on a PCA plane constructed using healthy and GRMD adults as active individuals, the LRMD dogs projected in the GRMD cloud, attesting the gait characteristic similarities between both colonies (Fig. 3e).

##### Force measurement

**Fig. 4:**
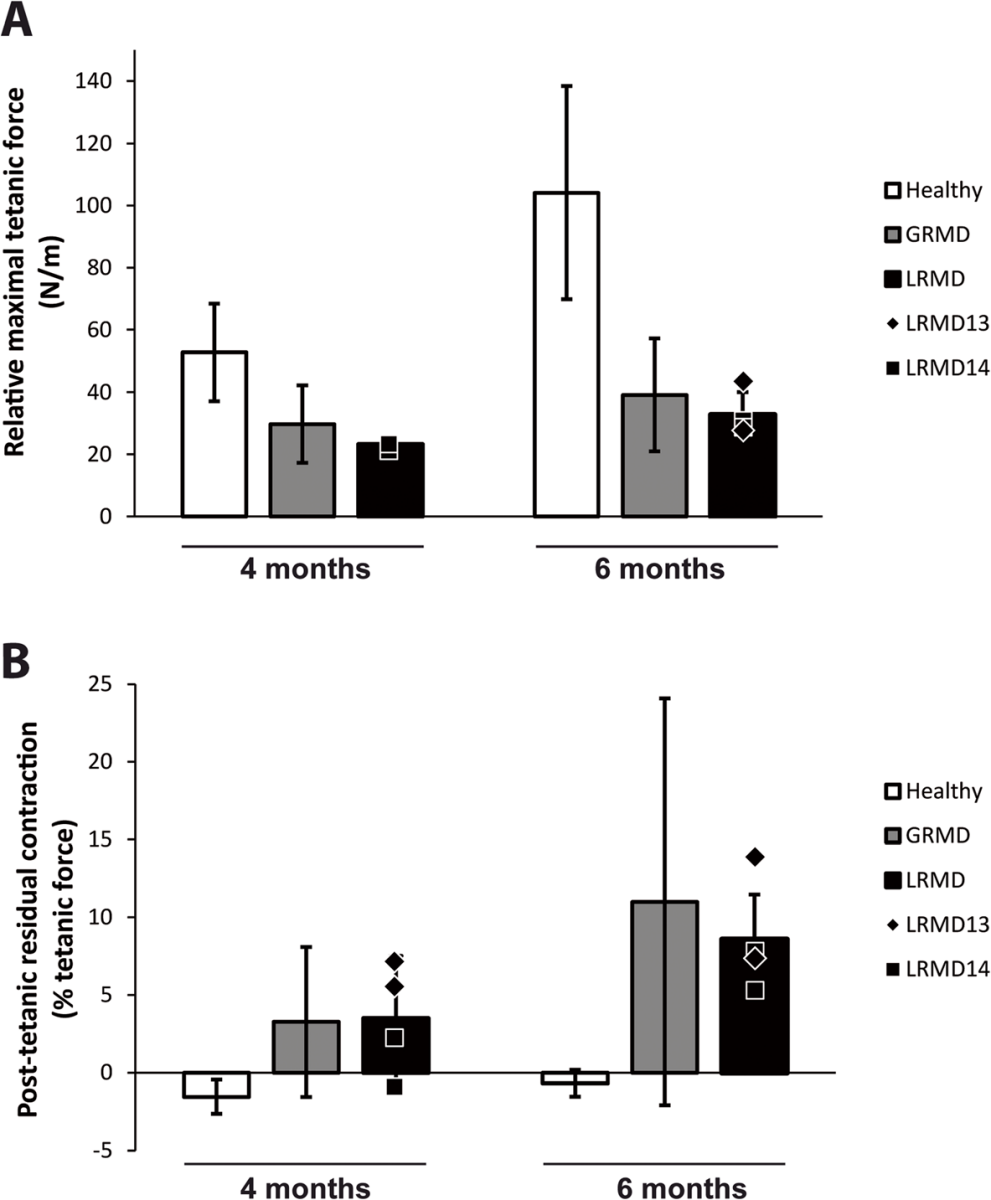
Force and relaxation measurement in LRMD dogs at 4 and 6 months of age. Histograms represent mean value ± SD of healthy (white), GRMD (grey) or LRMD (black) populations. The black symbols represent the individual values of the two LRMD dogs studied (2 pelvic limbs measured for each animal). **a** Relative maximal tetanic force, at 4 and 6 months of age, obtained after normalisation by the leg length in order to account for differences in dog size. Muscle force was decreased in LRMD dogs compared to healthy dogs, and it was slightly reduced compared with GRMD dogs. **b** Post-tetanic residual contraction as a percentage of the tetanic force at 4 and 6 months of age, was measured by normalising the difference between pre- and post-tetanic baselines by the maximal tetanic force. An incomplete relaxation after a tetanic stimulation was observed in LRMD dogs as well as in GRMD dogs, with an increase of this relaxation defect with age

Two dogs were subjected to muscle force tests, at 4 and 6 months of age. Muscle force was decreased compared to healthy dogs. The decrease in force observed in these animals was slightly higher than the one observed in GRMD dogs (Fig. 4a). Muscle relaxation impairment is a feature of GRMD, showcased by the presence of incomplete relaxation after a twitch or a tetanic contraction; these muscle relaxation impairment signs are variable and usually correlate with the severity of the phenotype. This feature was also found in LRMD dogs, with an increase of residual post-tetanic contraction at the age of 6 months (Fig. 4b).

##### Respiratory function

**Fig. 5:**
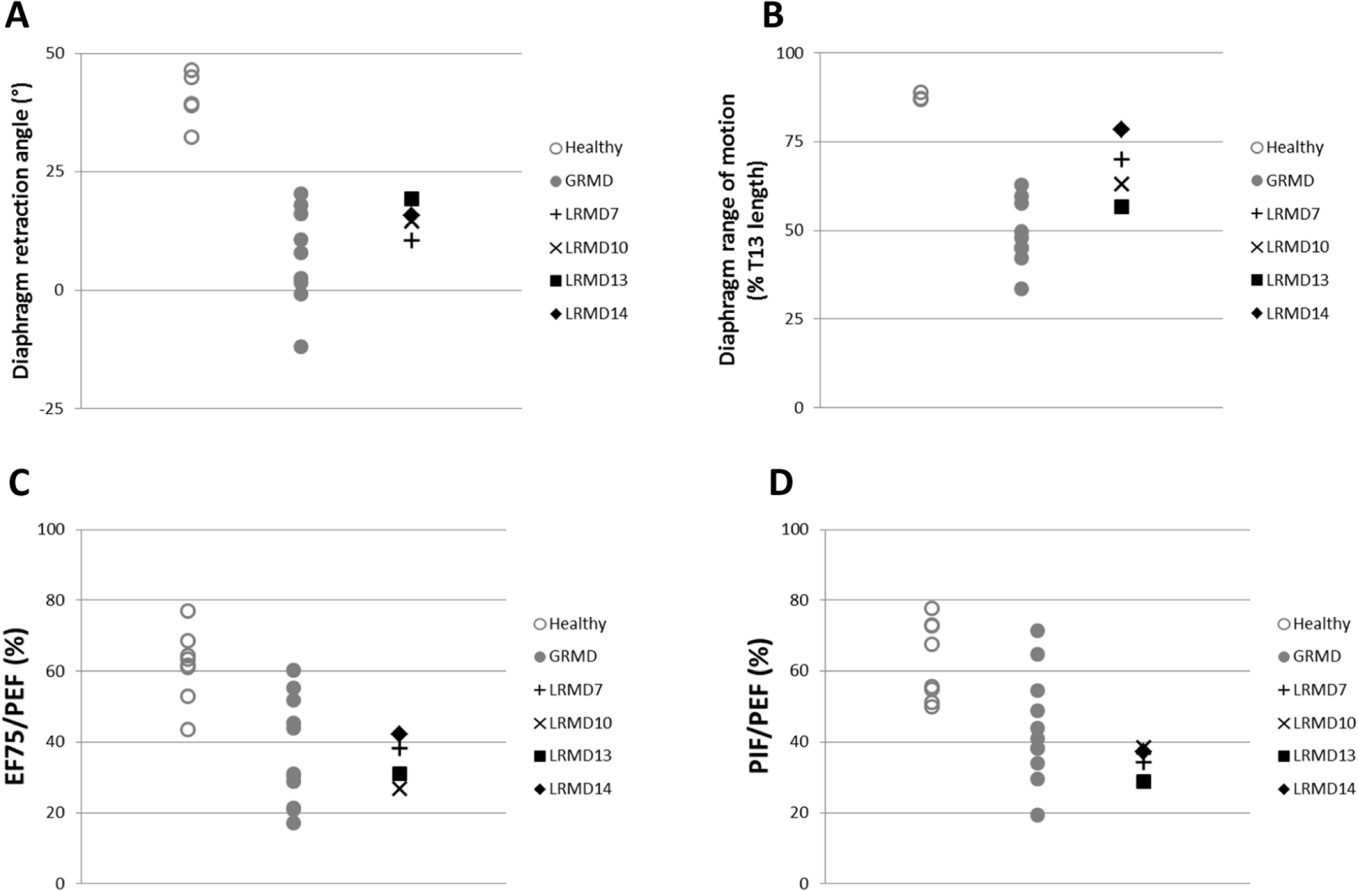
Respiratory function in LRMD dogs. Healthy dogs are represented by empty grey circles, GRMD dogs by grey dots, and LRMD dogs by black symbols. Dogs included in this assessment were adults (> 10 months). **a**, **b** Results of diaphragmatic kinematics on videofluoroscopic acquisitions. **a** The angle formed at the ventral edge of the diaphragmatic foramen of the caudal vena cava, between a line perpendicular to the vertebral axis and a line joining the caudal edge of the 11th thoracic vertebra is reduced in LRMD dogs attesting to the caudal retraction of the diaphragm at similar levels as GRMD dogs. **b** The diaphragm range of motion is decreased in LRMD dogs compared to healthy dogs, but to a lesser extent than in GRMD dogs. **c**, **d** Results from spirometric acquisitions. **c** The expiratory flow at 75% of the expired volume, expressed as a percentage of the peak expiratory flow, is decreased in LRMD dogs, in comparison to healthy dogs; values observed in LRMD dogs overlap with those obtained in GRMD dogs. **d** The ratio of the peak inspiratory flow on the peak expiratory flow is decreased in LRMD dogs, overlapping with the lowest values obtained in the GRMD population. Interestingly, for most of the respiratory indices assessed, LRMD14, the dog with a milder locomotor phenotype, had better values than the other LRMD dogs studied. *T13* 13^th^ thoracic vertebra. *EF75* expiratory flow at 75% of the expired volume, *PEF* peak expiratory flow, *PIF* peak inspiratory flow

Four adult LRMD dogs underwent respiratory tests and showed, as observed for other physiological functions, a similar pattern to the one seen in GRMD dogs. LRMD dogs showed a significant caudal retraction of the diaphragm measured by the angle index (*p* < 0.001); the retraction observed was similar to the one observed in GRMD dogs (no significant difference between both models) (Fig. 5a). The LRMD diaphragm was hypokinetic, with a decreased range of motion (*p* = 0.005), but slightly less affected than that of GRMD dogs (*p* = 0.02) (Fig. 5b). The flow-volume loop analysis showed a dramatic flow decrease at the end of expiration as indicated by a decrease of the EF75/PEF ratio *(p* = 0.0004); statistically significant differences were found for this parameter between both DMD models (Fig. 5c). The PIF/PEF ratio was also dramatically decreased (*p* < 0.0001), as it was the case for GRMD dogs (no significant difference between both models), compared to healthy animals. It should be noted however, that the LRMD dogs tended to have ratios that overlap with the lowest values observed for GRMD dogs (Fig. 5d).

##### Echocardiographic findings

A dog examined at a young age (LRMD4, 5 months) exhibited hyperechoic lesions on the left ventricular free wall, but the measures performed using conventional echocardiography were within normal range (shortening fraction 47.5%). However, tissue Doppler imaging, used to analyse the radial motion of the left ventricular free wall, revealed a slightly decreased endo-epicardial gradient of velocity, a hallmark of early pre-symptomatic dilated cardiomyopathy; this alteration has also been reported in GRMD dogs [54]. Another dog examined at the age of 5.5 and 7.5 years (LRMD7) showed dilated cardiomyopathy with a marked decrease of the shortening fraction worsening with time (21.5% at 5.5 years of age and 10.3% at 7.5 years of age). This dog also showed frequent ventricular arrhythmias (left and right ventricular premature contractions), which were suspected to be the underlying cause for the sudden death of this animal 1 year after the last echocardiographic examination.

### Identification of the causal mutation

**Fig. 6:**
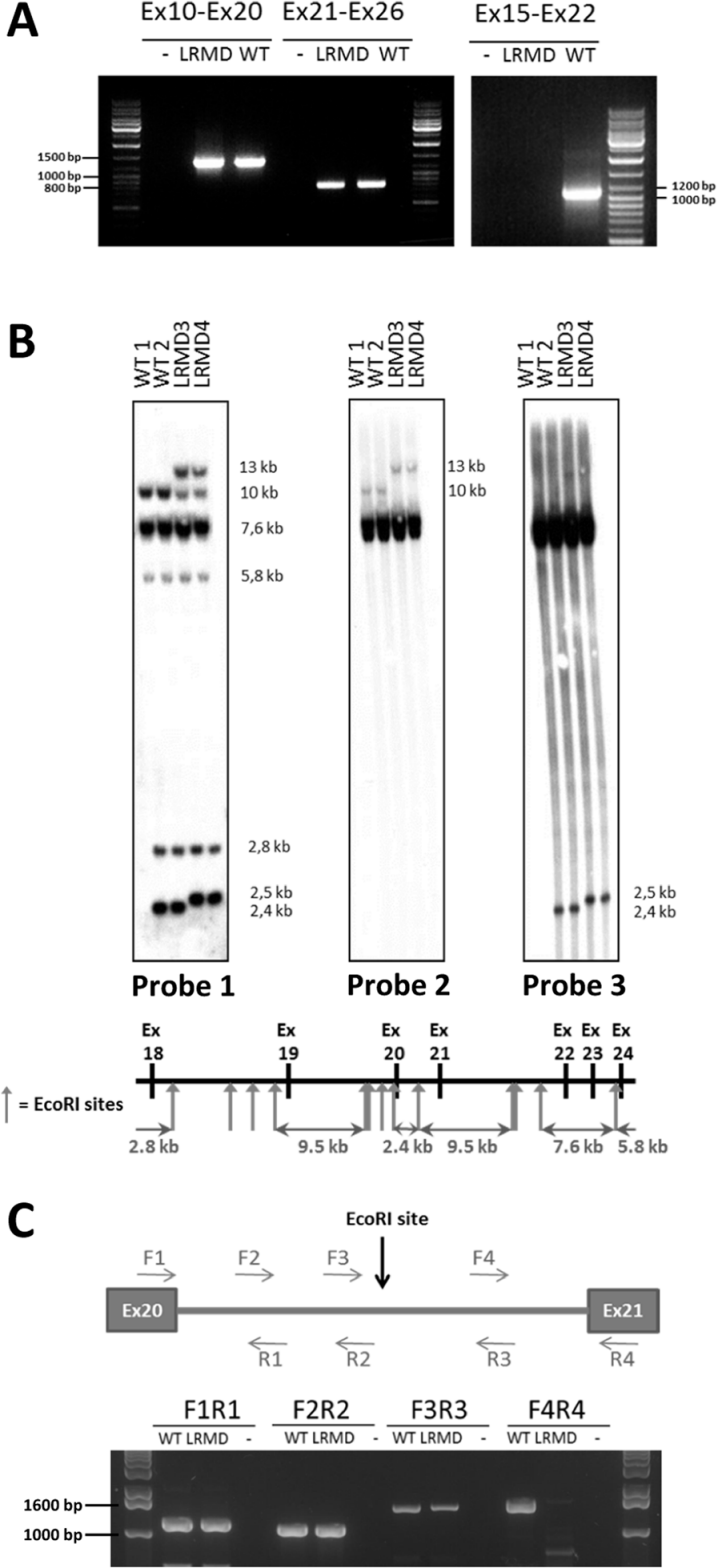
Mapping of the LRMD mutation. **a** Nested RT-PCR on the *DMD* cDNA. Normal-sized amplicons were obtained from the cDNA isolated from LRMD dogs, including the amplicons obtained using primers for exons 10 and 20 (expected size 1373 bp) and exons 21 and 26 (expected size 807 bp). A RT-PCR using primers to amplify exons 15 to 22 yielded a normal-sized amplicon (1095 bp) in the WT dog, but no band in the LRMD dog. **b** Southern blot on the EcoRI digested gDNA from two WT Labrador dogs unrelated to the LRMD colony and two LRMD dogs. The EcoRI restriction map of this region is schematized below the southern blot. The digestion profile of samples obtained for two LRMD dogs revealed by Probe 1, covering exons 18-24, differed from the one obtained from healthy animals. In the LRMD restriction map, an extra-band, around 13 kb, and a 2.5 kb band replacing the 2.4 kb wild-type band were observed. The use of Probe 2 covering exon 21 showed an abnormal band at 13 kb that had shifted from its normal size at 10 kb. The use of Probe 3 covering exon 20 showed an abnormal band at 2.5 kb; this band had shifted from its normal size at 2.4 kb. These results suggest that a gross rearrangement in intron 20 had occurred. **c** Exploration of intron 20 (4.5 kb in WT dogs) in a LRMD and a WT littermate by PCR, using 4 overlapping reactions. Normal size bands were obtained using the first three pairs of primers. The last pair of primers, designed to explore a 1661 bp region including the junction between intron 20 and exon 21, downstream the EcoRI restriction site, failed to amplify any product in LRMD dogs, even when using long-range PCR conditions

Overlapping RT-PCRs covering the dystrophin cDNA led to the amplification of all regions of the *DMD* transcript in LRMD dogs, with the exception of the sequence encompassing exons 20 and 21. Indeed, RT-PCRs between exons 10 and 20 and between exons 21 and 26 amplified normal products while no product could be obtained between exons 15 and 22 (Fig. 6a). The presence of a normal size exon 10-20 RT-PCR amplicon ruled out the possibility that the LRMD dogs carried the same mutation as the one reported in another strain of Labrador Retrievers (pseudoexon inserted in the intron 19 leading to a premature stop codon) [6, 37].

A Southern blot of the EcoRI-digested genomic DNA of LRMD dogs and using cDNA probes encompassing exons 18–24, exon 21 or exon 20 revealed abnormal bands (Fig. 6b), confirming a putative remodelled spot at the DNA level, mapped between exons 20 and 21 of the *DMD* gene.

The complete intron 20 (4.5 kb) was thereafter explored by PCR using four overlapping couples of primers (Fig. 6c). The three first pairs covered the 3.2-kb 5′ segment of intron 20; the PCR products amplified using these primers were of the expected size. By contrast, the fourth PCR using the F4R4 primers yielded no amplicon in LRMD dogs (Fig. 6c), pinpointing that a sequence remodelling had occurred in the 1.6 kb of the 3′ region of the intron. New primers designed to amplify the putative mutation site allowed to correctly amplify the 640 bp segment covering the 3′ end of the intron; subsequent sequencing of the amplicon confirmed a normal acceptor splice site in LRMD dogs. However, a 700 bp region (X: 27,623,229 to X: 27,622,528) remained non-amplifiable even when using long-range PCR conditions. Altogether, these molecular data confirmed a gross genomic DNA rearrangement involving the nearly 3′ end of intron 20. In addition, when compared to a healthy unrelated Labrador Retriever, a slight size difference was seen in the amplicon using the F1R1 pair of primers, which appeared to be longer in dogs belonging to the LRMD colony regardless of their clinical status (data not shown). Sanger sequencing showed a normal donor splice site in LRMD dogs, but revealed an insertion of 36 bp, 960 bp downstream the 5′ end of intron 20, including a 21 bp polyA and a repetition of 15 bp of the adjacent normal sequence. This insertion explained the shift in size of the smallest band on the southern blot using the exon 20 probe (Fig. 6b). We identified this insertion in all animals of the colony, including healthy dogs and thus concluded that this insertion was a DMD-unrelated polymorphism that segregates in this line of Labradors.

**Fig. 7:**
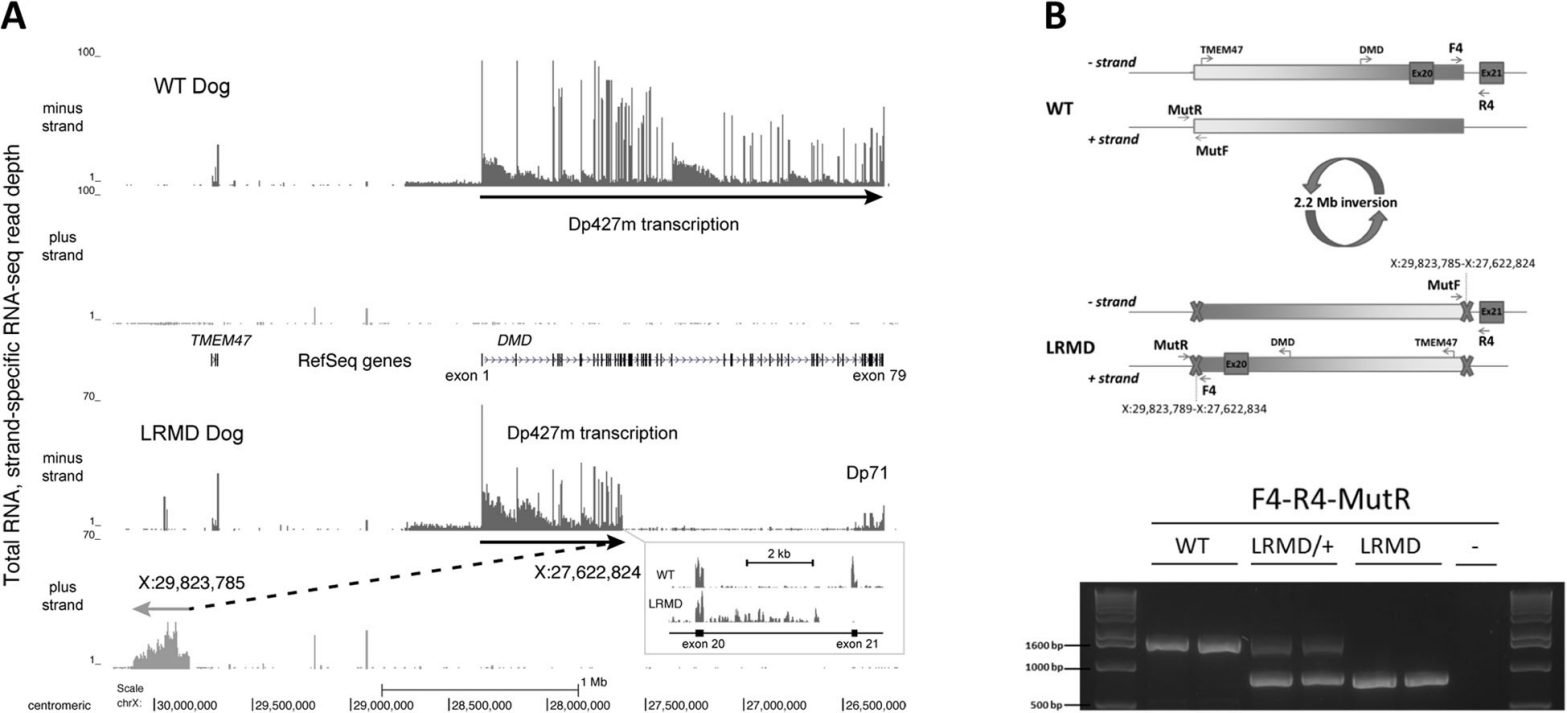
RNA-seq-based identification of the LRMD mutation. **a** A 4 Mb region spanning canFam3 chrX:30,200,000–26,200,000 is shown with RNA-seq read depths observed in a *biceps femoris* biopsy experiment plotted with RefSeq gene annotations. The upper panel shows Dp427m minus-strand transcript levels (*DMD* exons 1 through 79) seen with an unaffected dog, while the lower panel shows the inversion inferred from minus-strand *DMD* transcription and plus-strand ectopic transcription seen with LRMD8. The boxed inset shows a detailed view of intron 20 transcription from these two animals. **b** Schematic representation of the LRMD mutation, and LRMD genetic diagnosis by PCR. Two pairs of primers were used: the first set (F4R4) amplifies a 1661 bp product in the WT dog but not in the LRMD dog (breakpoint between these two primers). A second pair of primers (MutFMutR) was designed to amplify the distant site across the breakpoint yielding a 1939 bp product in the WT sample but not in the LRMD (data not shown). The proposed diagnostic PCR test relies on the use of F4 as a forward primer, and the combination of R4 and MutR as reverse primers to amplify either the WT or the LRMD allele. Using this test, a unique PCR product around 1600 bp was observed in the samples obtained from the LRMD healthy littermates (amplicon including F4-R4); a unique PCR product around 900 bp was observed in the samples of the two affected LRMD dogs (amplicon including F4-MutR); and both amplicons were observed in samples obtained from carrier females

The LRMD-causing mutation involving the 3′ region of intron 20 was then studied by RNA-sequencing with the aim of quantifying *DMD* transcription levels from both exons and introns using strand-specific libraries prepared from total RNA. This experiment revealed that the minus strand transcription from the Dp427m promoter abruptly decreased within the F4–R4 PCR region located between exons 20 and 21 (Fig. 7a). Ectopic transcription was observed in a region normally located 2 Mb farther away, towards the centromere on the plus strand 100 kb proximal to the *TMEM47* gene, where this novel transcription continued for ~ 0.3 Mb (X: 29,824,000–30,122,000) into a region of the X chromosome containing no annotated element. The level and pattern of transcription across this ~ 0.3 Mb region was similar to *DMD* large introns, suggesting that the causal mutation in LRMD dogs may be a 2.2 Mb inversion disrupting the *DMD* gene and involving *TMEM47* (Fig. 7a).

In light of these results, a new PCR experiment was designed with the aim of sequencing the breakpoint flanking regions, and developing a LRMD mutation diagnostic test able to identify carriers, healthy and diseased dogs. A pair of primers (MutFMutR) flanking the presumed distant breakpoint was designed, and the absence of an amplification product when used in the LRMD genome confirmed the location of a breakpoint between these primers. The use of these primers in combination with the primers flanking the intron 20 breakpoint (F4R4) amplified a PCR product in LRMD but not in healthy dogs. The sequencing of these PCR products indicated that the LRMD mutation was a 2.2 Mb inversion of a region encompassing nucleotides 27,622,834 to 29,823,785 and which can be annotated: chrX:g.27,622,834_29,823,788 inv. This inverted region included the entire *TMEM47* gene without disrupting it. The sequencing of the two breakpoints revealed a 27,622,834 to 29,823,789 junction on the one hand (PCR F4-MutR), and a 27,622,824 to 29,823,785 junction on the other hand (PCR MutF-R4). This indicated that the inversion is associated with a 9 nt loss in intron 20 and a 3 nt loss in the distant region, most likely suggesting that the LRMD mutation probably occurred after two double strand breaks 2.2 Mb apart, followed by a process of DNA repair through non-homologous end joining after inversion of the released fragment. Based on PCR confirmation of the breakpoint from genomic DNA, close inspection of the RNA-seq data revealed several intronic reads that mapped across the 27,622,834 to 29,823,789 breakpoint and several spliced exonic reads that mapped from *DMD* exon 20 to multiple locations beyond the inversion breakpoint, confirming transcription and splicing from the *DMD* region. Finally, a multiplex PCR was designed to generate a genetic test able to reliably identify LRMD, carrier and healthy dogs, and we confirmed its ability to discriminate between the three genotypes (Fig. 7b).

### Characterisation of the dystrophin expression in muscles

**Fig. 8:**
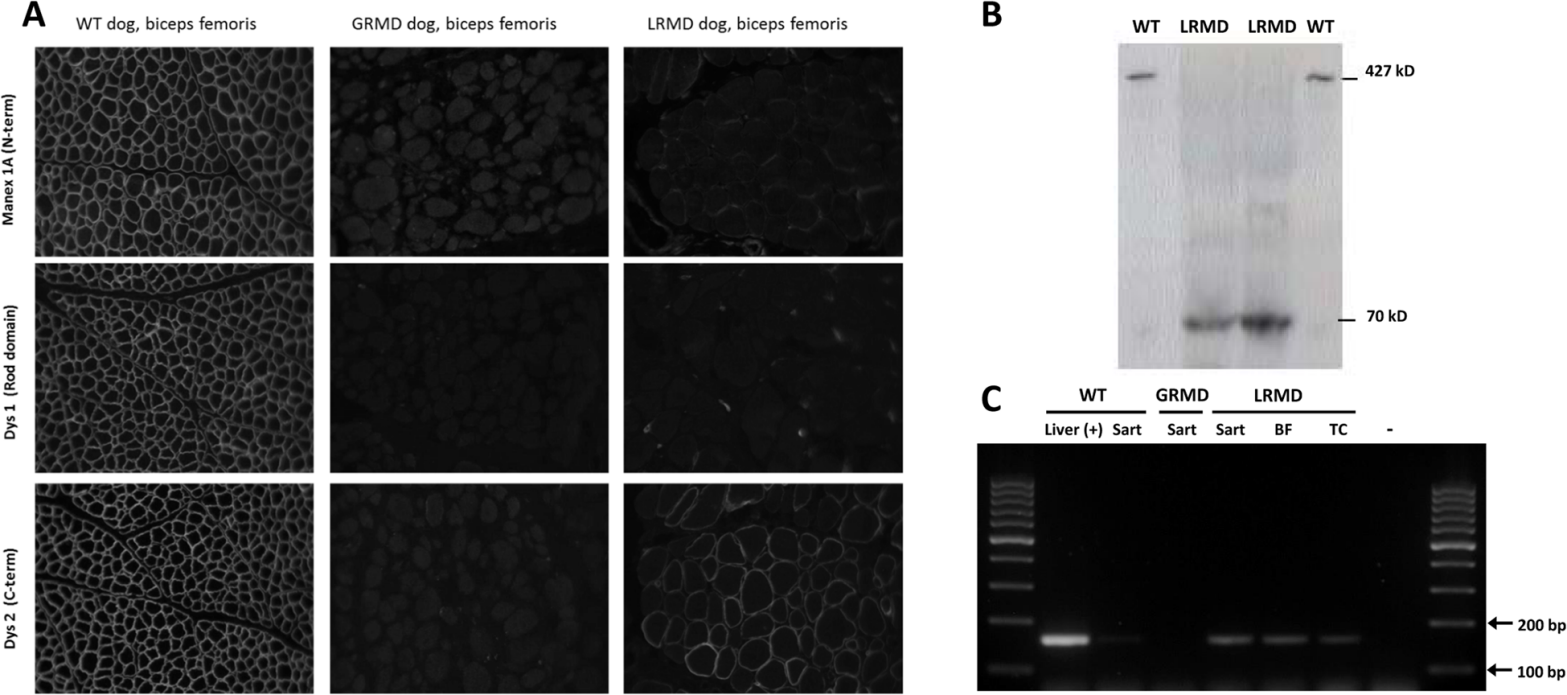
Expression of the Dp71 isoform in LRMD muscles. **a** Comparative immunohistochemistry analysis of biopsies from the *biceps femoris* of a healthy dog, a GRMD dog and a LRMD dog. Three antibodies were used: MANEX1A (N-terminal part), Dys1 (Rod domain, downstream of the mutation), Dys2 (C-terminal part). As expected, the staining was positive in the muscle of the healthy dog, and negative for the GRMD dogs regardless of the antibody used. The sample obtained from the LRMD dog was negative for MANEX1A and Dys1, and positive for Dys2, but the staining was found to be heterogeneous among fibres. **b** Western blotting using the Dys2 antibody (C-terminal part). Muscle protein extracts from two healthy animals were loaded in wells 1 and 4 (50 μg); a normal size band corresponding to the full-length dystrophin (427 kD) was seen in these samples. Muscle protein extracts from LRMD muscles (250 μg) were loaded in wells 2 (LRMD3, *biceps femoris*) and 3 (LRMD3, *interosseus* muscle); a band at around 70 kD was observed in these lanes indicating that the protein detected was truncated. **c** RT-PCR using a forward primer designed to bind at the junction between the specific first exon of the Dp71 and the exon 64, and a reverse primer at the junction between exons 65 and 66. cDNA from a canine liver was used as a positive control showing a band of the expected size (164 bp). A very faint band was observed using the cDNA from a muscle sample obtained from a healthy dog and no band was observed for the GRMD dog. Conversely, in the three samples obtained from LRMD muscles (LRMD8, *sartorius cranialis*, *biceps femoris*, *tibialis cranialis*) a band at the same size as the positive control, though less pronounced, was seen, indicating that the Dp71 transcript is present in LRMD muscle. *Sart sartorius cranialis* muscle, *BF biceps femoris* muscle, *TC tibialis cranialis* muscle

Dystrophin immunostainings performed on LRMD muscle biopsies using an antibody directed against the C-terminal part of the protein (Dys2) showed a faint but undoubtful signal with normal subsarcolemmal localization (Fig. 8a). The percentage of Dys2-positive myofibres was evaluated on 32 skeletal muscle biopsies from 7 LRMD dogs and was found to range from 0.2% (LRMD6, *sartorius cranialis* muscle) and 44.1% (LRMD1, *tibialis cranialis*), with a mean of 11.6% (SD = 12.3%). In order to better characterise this protein, monoclonal antibodies cross-reacting with different regions of the protein were used on serial sections. No Dys2-positive fibres were seen using any of the antibodies cross-reacting with upstream regions of the dystrophin protein: the staining was negative for the N-terminal part (MANEX1A), the first repeat of the rod domain (MANEX1011 C) or for parts of the rod domain located downstream of the mutation (Dys1, MANDYS107 (Figure S3)) (Fig. 8a). In some of the biopsies, a few fibres that were positive for the N-terminal part of the protein were found MANEX1A+ and MANEX1011C+; these fibres were however not recognised by the antibody specific for the C-terminal part of the protein (Dys2) (Figure S4).

In order to investigate the size of the LRMD N-terminal truncated dystrophin, a Western blot analysis was performed using the Dys2 antibody (C-terminal portion). The analysis of the two biopsies tested indicated the presence of a protein of around 70 kD (Fig. 8b). These results led us to speculate that the protein expressed in these muscles could be the Dp71 isoform. The expression of this isoform was confirmed with RT-PCR using a forward primer designed to bind the specific first exon of the aforementioned isoform (Fig. 8c); this result was consistent with RNA-seq coverage data observed in the Dp71 region (Fig. 7a). No expression of this isoform was found in a biopsy originating from a GRMD dog, suggesting that this Dp71 expression is not a compensatory mechanism in a context of canine dystrophinopathy, but rather specific of the LRMD context. Finally, we wanted to know if there was a correlation between the percentage of Dys2-positive fibres and the muscle lesions observed in the H&E stained sections. For this purpose, a correlation study was performed on 28 muscle biopsies and indicated that there was no correlation between the two phenomena (Pearson’s *R* = − 0.34, *p* = 0.069), suggesting that this isoform expression has no major influence on muscle pathology (Figure S4).

### Investigation of known disease modifiers: *Jagged1*, *Pitpna* and *LTBP4*

In order to asses if the inter-individual heterogeneity observed in the LRMD colony could be related to polymorphisms in known modifier genes, the presence of the described mutation in the *Jagged1* gene promoter leading to ‘escaper’ phenotypes in dogs [41] was investigated in 5 LRMD dogs with diverse phenotypes, including both the animal with the mildest phenotype (LRMD14) and the oldest survivor (LRMD7). The results showed that the sequence of this promoter was identical in all the LRMD dogs tested and that none of these dogs harboured the G>T point mutation described in GRMD escapers. Furthermore, the *Pitpna* mRNA expression levels in skeletal muscle were assessed in four LRMD dogs. The levels of *Pitpna* mRNA have been found to be decreased in escapers compared to severely affected GRMD dogs [42]. For this assessment, we used two severely affected animals (LRMD8 and 9) and the two less severely affected LRMD dogs (LRMD7 and 14). No differences in expression levels were seen among the studied dogs (*p* = 0.921), suggesting that the phenotype heterogeneity is not associated with fluctuations in *Pitpna* mRNA expression in LRMD dogs.

Polymorphisms in the coding sequence of *LTBP4* have been associated with modulation of the phenotype in *mdx* mice and in human DMD patients [61, 62]. Eleven SNPs were found in the canine *LTBP4* coding sequence, 4 of these SNPs were responsible for amino-acid changes (amino acids number 425, 439, 545 and 668). The study of these polymorphisms in 5 LRMD dogs showed that all the animals were homozygous and identical for each of the SNPs. Particular attention was put on the 4 SNPs that could potentially lead to amino acid changes. The results obtained showed that amino acid 425 was a Threonine, 439 and 545 were Prolines and 668 an Alanine. In conclusion, the analysis performed showed that none of the modulators analysed were responsible for the inter-individual heterogeneity. The homozygosity observed in the LRMD colony is most likely related to the high inbreeding rate of the colony; this fact probably makes this LRMD model a favourable context to discover new DMD phenotype modifiers.

## Discussion

This study provides a full characterisation of a new canine model of Duchenne muscular dystrophy, named LRMD, in a Labrador Retriever strain. Several descriptions of dystrophin deficiencies have been reported in the canine species up to date (Table S2). The originality of the present study relies in the direct comparison of this novel model with the ‘reference’ canine model, the GRMD dog, in order to identify potential interests of the LRMD model. A comparison to other canine models will also be discussed and it is summarised in Table S2.

Clinical, histological and functional findings were comparable to those usually seen in the GRMD model. Interestingly, the evaluation tools developed for the GRMD model were completely transposable to the LRMD and indicated that the values of the parameters assessed largely overlap in both models. These tools can thus be used in other dog models, to characterise a muscular dystrophy phenotype. Some of the parameters measured indicate that there is a trend towards a slightly more severe phenotype in LRMD dogs. This finding might be related to the high inbreeding rate of the colony; a factor that has previously been reported to be a disease severity enhancer in dogs [6]. The severity of the LRMD disease described here makes these LRMD dogs strikingly different, from a phenotypic point of view, from another strain of dystrophic Labradors described elsewhere that have shown to be almost asymptomatic, for which the mutation remains unknown [27]. A 184 bp insertion in intron 19 has also been reported in another Labrador Retriever strain. This model has not yet been extensively described from a phenotypic standpoint but seems likely to exhibit a classical GRMD-like phenotype, closer to the one observed in our LRMD dogs [6, 37].

The strong inter-individual heterogeneity of the GRMD model reported by many groups makes the use of alternative models attractive. Consequently, special attention was paid to this specific aspect in LRMD dogs. As mentioned previously, the colony studied here presented a high degree of inbreeding among the individuals; this feature could have accounted for the higher phenotypic homogeneity observed between 2 and 4 months. Otherwise, the inter-individual heterogeneity found in the LRMD colony was comparable to the one seen in GRMD dogs. After the age of 4 months, functional evaluation showed a marked divergence among animals, from relatively mildly affected dogs that maintained ambulation, to dogs with severe ambulation disability. This latter phenotype was very similar to the so-called ‘severe forms’ of GRMD described in the French colony that are associated with an early loss of ambulation [40].

In order to address the underlying cause of the inter-individual heterogeneity found in this colony, we focused on two of the previously described DMD modifier genes: *Jagged1*, described in dogs [41] and *LTBP4* identified in DMD patients [61]. The analysis of these modifier genes indicated that no modifications had occurred that could explain the phenotype variability seen in LRMD dogs. Differential expression of *Pitpna* has also been described to vary according to phenotype severity in GRMD dogs [42]. Our results showed that this variability was not present in our colony of LRMD dogs and could consequently not be associated to the inter-individual heterogeneity observed in these animals. This could be explained by the fact that *Pitpna* might be downregulated only in rare escaper dogs that were not found in the LRMD colony. The existence of a wide inter-individual heterogeneity in a context of a relatively homogeneous genetic background, as attested by homozygosity in *LTBP4* SNPs, and as a consequence of the high inbreeding rate, makes this LRMD colony a unique opportunity to identify new modulators of the disease in a facilitated genetic context.

The low number of examined dogs is one of the weaknesses of this descriptive study. This is particularly relevant for some functional tests that would probably require a follow-up study with additional dogs to obtain a complete natural history, as a prerequisite for using this model in therapeutic trials. The low number of animals used in some of the functional assessments was due to the fact that the development of the evaluation tools occurred at the same time as the LRMD colony was established. Another limitation of the present study was that LRMD animals belonging to both sexes were used while only male GRMD were studied. In order to compensate for the low number of animals, we chose to include in the study functional data obtained from female LRMD dogs; attention was paid to normalise all the assessments used in this study that were potentially impacted by animal size. However, some reports have pointed out that female dystrophin-deficient dogs may be less affected than males [5]. In the present study, inter-individual heterogeneity was seen among the 5 affected females, although it should be mentioned that both the mildest clinical form (LRMD14) and the oldest survivor (LRMD7) were females.

Another area of optimisation not currently covered by the GRMD model would be presence of mutations that are relevant to the human condition. Most human cases of DMD are due to large mutations, predominantly deletions, preferentially affecting the major mutation hotspot spanning exons 45 to 55 or to a lesser extent the minor hotspot (exons 2–20) [63]. Duplications also account for a significant number of the identified disease-causing mutations; exon 2 duplications are particularly frequent [63, 64]. The GRMD mutation is a splice site point mutation and is therefore not a model for the large mutations that are frequent in DMD patients. Splice site mutations however account for 3% of the DMD referenced mutations. The LRMD mutation is a gross rearrangement, but it should be mentioned that inversions are a rare cause of human DMD. Querying the Leiden Duchenne muscular dystrophy database provided 8 inversion entries [65] and 5 supplementary inversion reports have been found in the literature [66–70]. As it is the case of the GRMD, the LRMD is thus an additional model that represents less frequent mutational phenomena leading to DMD in humans. Alternatively, the Cavalier King Charles dog that carries a splice site mutation in intron 50 is amenable to exon 51 skipping, a therapeutic approach relevant to approximately 14% of the DMD patients and is therefore considered a more relevant model from a genetic point of view; this model was recently successfully used in a gene therapy study [24, 48, 63].

The mutation present in our LRMD colony would not be amenable to exon skipping therapy because of the complexity of the genetic rearrangement. However, this mutation could serve to model complex/large mutations that would not benefit from exon skipping strategies but rather, for example, from gene replacement strategies. In this context, the LRMD model can probably be considered naïve, from an immune point of view, to a portion of the protein spanning exons 21 to 30 (beginning of the Dp260 transcription, presumably preserved). This context is different from the one of the GRMD dog, in which revertant fibres express a protein lacking only exons 3 to 9 or 5 to 12 [71]. Both GRMD and LRMD models are therefore not optimal to model immune response questions in the context of most common human mutations. The German Shorthaired Pointer muscular dystrophy dog (GSHPMD) in which a deletion of the whole dystrophin-gene occurred, as well as the more recently described Miniature Poodle are undoubtedly good models for such investigations [17, 36].

The GSHPMD deletion not only involves the whole dystrophin gene but also the *TMEM47* (Transmembrane protein 47) gene. The GHSPMD dog is, consequently, a canine spontaneous ‘KO’ not only for the dystrophin gene but also for the *TMEM47* gene. This model does not exhibit other clinical signs in addition to those related to muscular dystrophy [36]. In LRMD dogs, the *TMEM47* gene is not disrupted but inverted, hence the transcription of this gene could be perturbed; this should be checked in future studies. *TMEM47* encodes for a highly conserved transmembrane claudin-like protein implicated in transition from adherent to tight junctions, and is highly expressed in several tissues including the foetal and adult brain [72, 73]. It has therefore been suspected to be implicated in some cases of X-linked mental retardation. A screening involving 16 affected patients revealed no *TMEM47* implication [74], but a case of *TMEM47* disruption has been linked to intellectual disability and language delay [73].

This 15th reported mutation in canine *DMD* gene was identified using RNA-seq. This method shows arousing interest in the field of mutation characterisation in undiagnosed rare diseases and particularly neuromuscular diseases. Even in the era of whole genome sequencing (WGS) and whole exome sequencing (WES), a significant amount of patients actually still suffer rare diseases for which the underlying mutation is yet to be identified. RNA-seq has been recently proposed as a good tool to help detecting mutations in these patients and appears to be particularly powerful in cases of mutations affecting gene splicing that are not detected by WES and WGS [75, 76].

The mutation reported here is the second inversion causing Duchenne-type muscular dystrophy in dogs. The other was described in a Japanese Spitz strain and it disrupts the *DMD* gene within intron 19, involves the *TMEM47* gene and disrupts the retinitis pigmentosa GTPase regulator (*RPGR*) gene [30]. Interestingly, in this comparable genetic context, the Dp71 isoform was also expressed in muscle tissue [30]. This isoform is normally expressed in non-muscle tissues, and its expression is downregulated during myogenesis. It has been demonstrated that two transcription factors, Sp1 and Sp3, bind to the Dp71 promoter activating transcription of this short isoform and that the absence of Dp71 in mature muscle fibres results from the repression of its expression by MyoD [77], thus favouring the presence of Dp427m isoform in the sub-sarcolemmal region. These unique examples of spontaneous expression of Dp71 in adult muscle as a result of the inversion of the proximal end of the dystrophin gene suggest that the Dp427m promoter could play a role in regulating Dp71 promoter repression. This regulatory role would be responsible for maintaining the Dp71 promoter activated because of a lack of control from the inverted proximal end of the dystrophin gene. Expression of the smaller Dp71 isoform, composed of the C-terminal part of the Dp427, allows for the correct localization of members of the dystrophin-associated protein complex, but Dp71 lacks actin-binding domains and is therefore nonfunctional in skeletal muscle. This phenomenon has been confirmed in the *mdx* model, where its expression in muscle tissue does not provide benefit and is even suspected to be deleterious [78–80]. This observation is in line with our findings, as the presence of this isoform in our model shows no significant impact on muscle pathology. The phenotypes of LRMD and GRMD dogs are comparable, but there was a trend towards an increased severity of symptoms in LRMD dogs, attesting to the lack of functional advantage provided by the Dp71 isoform and maybe even pinpointing a possible deleterious effect. One of the questions that remains to be answered is whether the expression of Dp71 could be counteracted, in this particular model, by a therapeutic fully functional protein, since some competition may exist between both proteins at the sub-sarcolemmal region.

## Conclusions

This study provides a comprehensive description of a new canine dystrophinopathy covering from molecular characterisation to quantitative phenotypic description. We have also performed a thorough comparison with the GRMD dog model, the most commonly used model in preclinical trials for DMD. Both diseases were phenotypically comparable, including inter-individual heterogeneity, and no major advantage of the LRMD model over the GRMD one could be found, for a use in preclinical therapeutic trials. Moreover, the particular mutational and transcriptional context found in the LRMD dog could be confusing in a context of therapeutic trials intending to bring clear responses about DMD treatment options. This model could however be of interest to better understand the mechanisms involved in the transcriptional regulation of dystrophin isoforms. Most importantly, the LRMD colony displays phenotype heterogeneity in a more homogeneous genetic background than the GRMD model. This feature, taken together with the fact that the LRMD model is a large animal model that faithfully reproduces DMD disease course, may be an advantage when attempting to identify and validate disease modulators.

## Supplementary information

**Additional file 1: Figure S1.** Pedigree of the LRMD colony. The colony was founded by crossing an affected male with his carrier sister. Ten subsequent litters were obtained, and a total of 14 LRMD dogs survived the neonatal period. The colony presented high levels of inbreeding (consanguinity coefficients min 25 % max 41 %).

**Additional file 2: Figure S2.** Phenotype in the neonatal period: fulminating form and growth retardation. A: Serial sections of muscle tissue sampled on a deceased LRMD neonate stained with H&E (left) and Alizarin red S (right)., Necrosis and calcium overload were seen in the diaphragm, and to a lesser extent, in the tongue and the *sartorius cranialis* muscle. Other muscles, such as the *triceps brachii*, the *tibialis cranialis* or the *biceps femoris,* were relatively spared by this rhabdomyolysis process. The results observed are consistent with the muscle lesions observed in the GRMD neonatal fulminating form. B: Whole section view of a diaphragm biopsy taken from a deceased LRMD neonate. The strong staining with Alizarin red S indicates that there is a significant calcium overload in this muscle. C: Kaplan-Meier survival curve during the neonatal period (from birth to 2 months of age) showing that half of the LRMD dogs died within their first days of life due to neonatal fulminating forms. Only 47 % of the LRMD dogs survived to weaning (2 months of age). D: Weight curves during the neonatal period (from birth to weaning, 2 months of age) from 5 different litters, showing growth retardation in most LRMD dogs (in black) relative to healthy littermates (in grey). E: Picture of a 15-week-old LRMD dog (LRMD7, on the right) compared to a healthy littermate (carrier female) illustrating the difference in size F: Picture of a one month-old LRMD dog (LRMD13, on the right) compared to a healthy male littermate.

**Additional file 3: Figure S3.** Histological findings in LRMD skeletal muscles. A: evolution of the muscle pathology with age. H&E stained biopsies x20. Illustration of the aspect of the *biceps femoris* at 5 different ages: 2 months, 4 months, 1 year, 2 years and 6 years. A significant number of necrosis-regeneration lesions are noted at early stages; these lesions are associated with inflammatory foci and sporadic calcifications. With time endomysial fibrosis and adiposis dominate the pathological context. B: illustration of all the elementary lesions found in LRMD muscles. Entire section and details of an *extensor carpi radialis* biopsy taken at the age of 4 months (LRMD7). This biopsy had an elevated pathological index (62.5 %). Abbreviations: BF: *biceps femoris*.

**Additional file 4: Figure S4.** Immunohistochemical characterization of the expressed dystrophins in LRMD muscles. Serial sections from a *biceps femoris* (LRMD3), immunohistochemistry using the following antibodies: A: Dys2 (dystrophin, C-terminal part), B: βDG (beta-dystroglycan), C : MANEX1A (dystrophin, N-terminal part), D : MANEX1011C (dystrophin, exons 10-11), E: Dys1 (dystrophin, central rod domain repeats 8-10), F: MANDYS107 (dystrophin, central rod domain repeat 15). Most of the myofibres show a marked immunoreactivity with the Dys2 (C-term) antibody, associated with a beta-dystroglycan relocalization. Some of the Dys2 negative myofibres (asterisks) were positive for the antibodies specific for the N-terminal part of the protein (MANEX1A, MANEX1011C). No immunoreactivity was seen in any case when using antibodies specific for the central rod domain.

**Additional file 5: Figure S5.** Correlation between Dp71 expression and histological lesions In 28 biopsies from 6 muscles sampled from 8 different LRMD dogs the proportion of Dys2+ fibres was quantified and compared to the pathological index on H&E stained serial sections. The correlation was not significant (Pearson’s R= -0.32; p =0.069).

**Additional file 6. Table S1**

**Additional file 7. Table S2**

## Data Availability

The datasets used and/or analysed during the current study are available from the corresponding authors on reasonable request.

## Abbreviations

DMD: Duchenne muscular dystrophy
GRMD: Golden Retriever muscular dystrophy
LRMD: Labrador Retriever muscular dystrophy
CK: Creatine kinase
PEF: Peak expiratory flow
PIF: Peak inspiratory flow
