# Supplementary figures and images for: X-linked muscular dystrophy in a Labrador Retriever strain: phenotypic and molecular characterisation
Source: Skelet Muscle. 2020 Aug 7;10:23. doi: 10.1186/s13395-020-00239-0 (PMC7412789; doi:10.1186/s13395-020-00239-0)

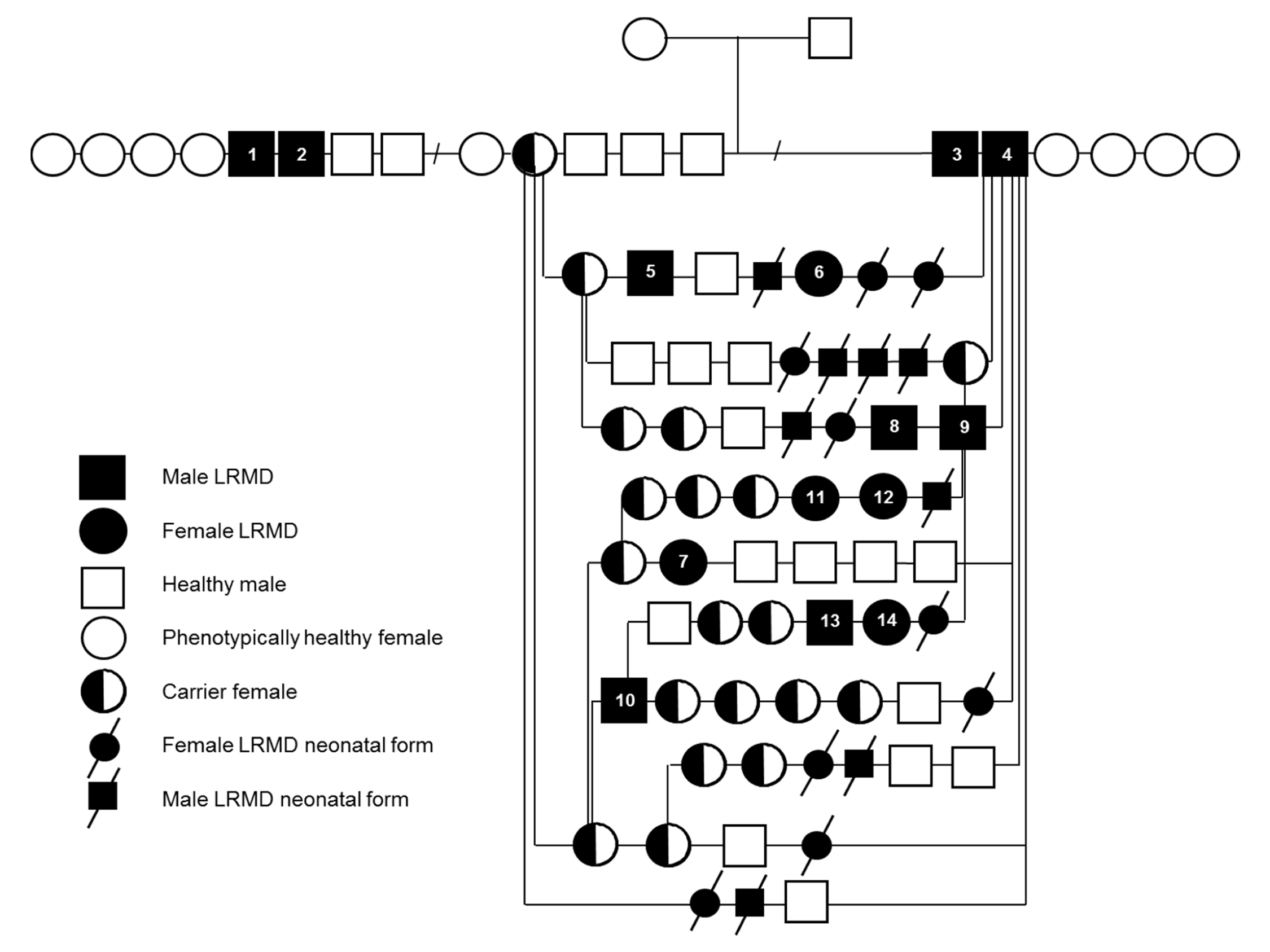

Supplement: Supplementary file 1 — Additional file 1: Figure S1. Pedigree of the LRMD colony. The colony was founded by crossing an affected male with his carrier sister. Ten subsequent litters were obtained, and a total of 14 LRMD dogs survived the neonatal period. The colony presented high levels of inbreeding (consanguinity coefficients min 25 % max 41 %). [file 13395_2020_239_MOESM1_ESM.tif]

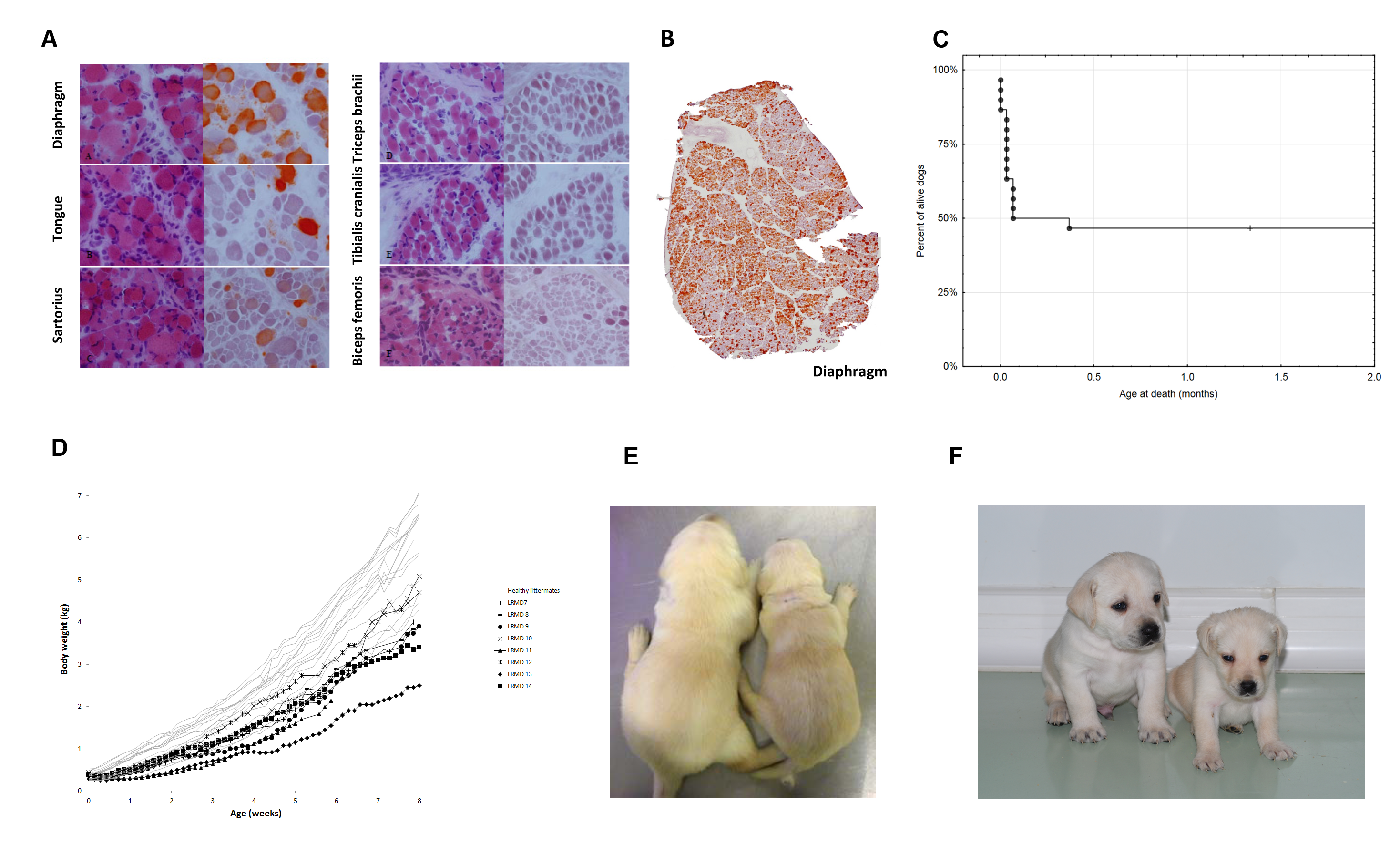

Supplement: Supplementary file 2 — Additional file 2: Figure S2. Phenotype in the neonatal period: fulminating form and growth retardation. A: Serial sections of muscle tissue sampled on a deceased LRMD neonate stained with H&E (left) and Alizarin red S (right)., Necrosis and calcium overload were seen in the diaphragm, and to a lesser extent, in the tongue and the sartorius cranialis muscle. Other muscles, such as the triceps brachii, the tibialis cranialis or the biceps femoris, were relatively spared by this rhabdomyolysis process. The results observed are consistent with the muscle lesions observed in the GRMD neonatal fulminating form. B: Whole section view of a diaphragm biopsy taken from a deceased LRMD neonate. The strong staining with Alizarin red S indicates that there is a significant calcium overload in this muscle. C: Kaplan-Meier survival curve during the neonatal period (from birth to 2 months of age) showing that half of the LRMD dogs died within their first days of life due to neonatal fulminating forms. Only 47 % of the LRMD dogs survived to weaning (2 months of age). D: Weight curves during the neonatal period (from birth to weaning, 2 months of age) from 5 different litters, showing growth retardation in most LRMD dogs (in black) relative to healthy littermates (in grey). E: Picture of a 15-week-old LRMD dog (LRMD7, on the right) compared to a healthy littermate (carrier female) illustrating the difference in size F: Picture of a one month-old LRMD dog (LRMD13, on the right) compared to a healthy male littermate. [file 13395_2020_239_MOESM2_ESM.tif]

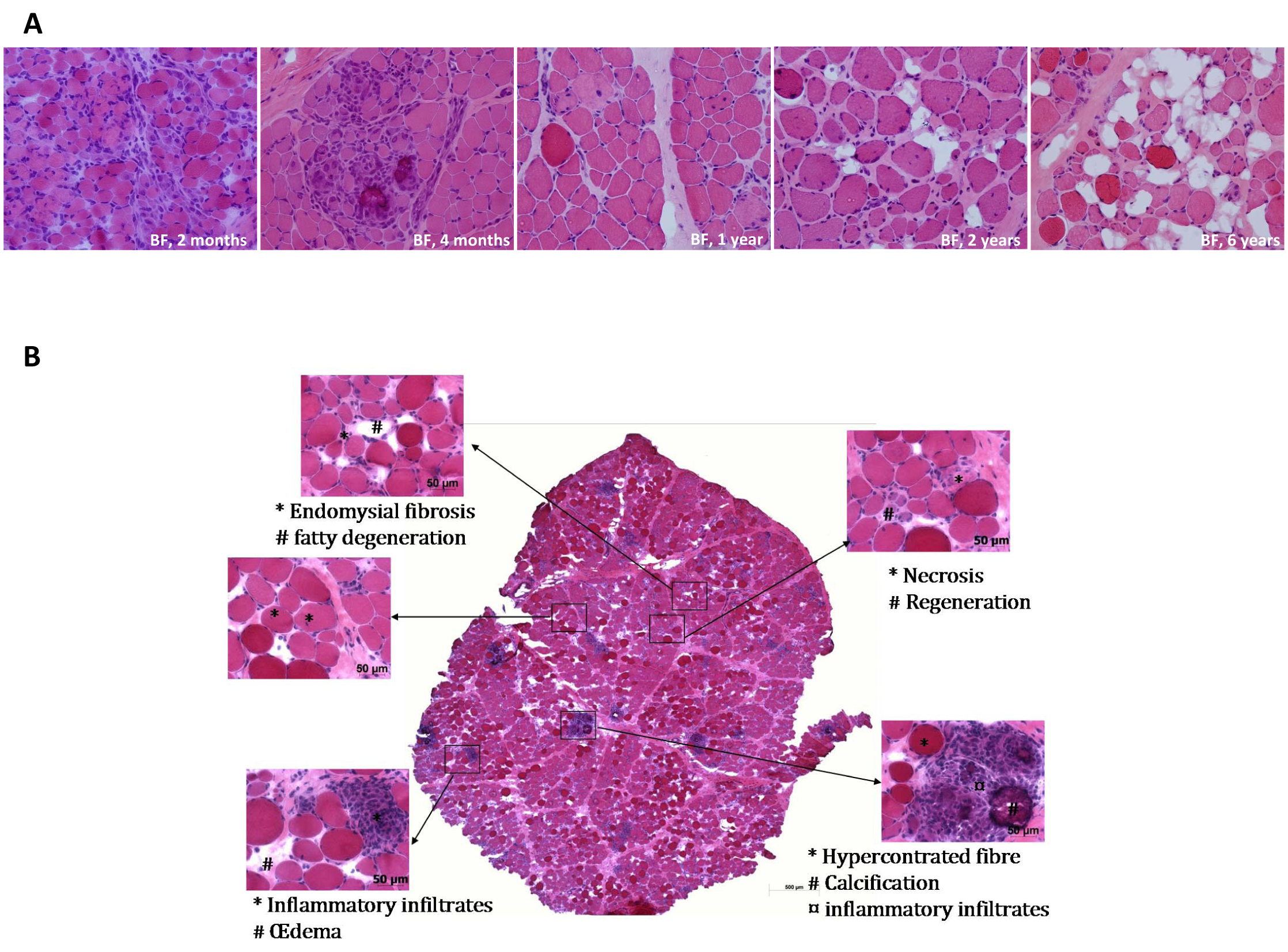

Supplement: Supplementary file 3 — Additional file 3: Figure S3. Histological findings in LRMD skeletal muscles. A: evolution of the muscle pathology with age. H&E stained biopsies x20. Illustration of the aspect of the biceps femoris at 5 different ages: 2 months, 4 months, 1 year, 2 years and 6 years. A significant number of necrosis-regeneration lesions are noted at early stages; these lesions are associated with inflammatory foci and sporadic calcifications. With time endomysial fibrosis and adiposis dominate the pathological context. B: illustration of all the elementary lesions found in LRMD muscles. Entire section and details of an extensor carpi radialis biopsy taken at the age of 4 months (LRMD7). This biopsy had an elevated pathological index (62.5 %). Abbreviations: BF: biceps femoris. [file 13395_2020_239_MOESM3_ESM.tif]

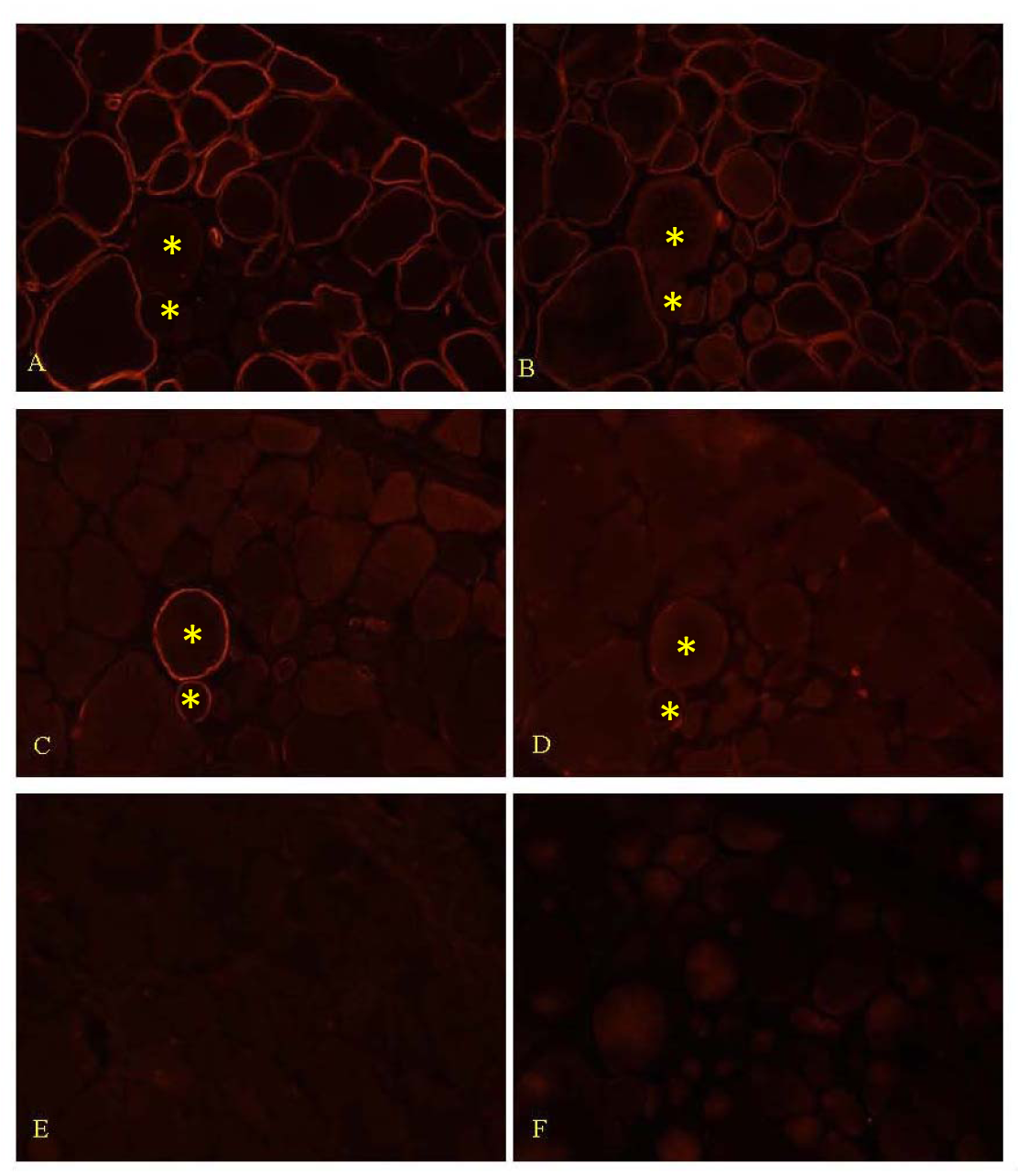

Supplement: Supplementary file 4 — Additional file 4: Figure S4. Immunohistochemical characterization of the expressed dystrophins in LRMD muscles. Serial sections from a biceps femoris (LRMD3), immunohistochemistry using the following antibodies: A: Dys2 (dystrophin, C-terminal part), B: βDG (beta-dystroglycan), C : MANEX1A (dystrophin, N-terminal part), D : MANEX1011C (dystrophin, exons 10-11), E: Dys1 (dystrophin, central rod domain repeats 8-10), F: MANDYS107 (dystrophin, central rod domain repeat 15). Most of the myofibres show a marked immunoreactivity with the Dys2 (C-term) antibody, associated with a beta-dystroglycan relocalization. Some of the Dys2 negative myofibres (asterisks) were positive for the antibodies specific for the N-terminal part of the protein (MANEX1A, MANEX1011C). No immunoreactivity was seen in any case when using antibodies specific for the central rod domain. [file 13395_2020_239_MOESM4_ESM.tif]

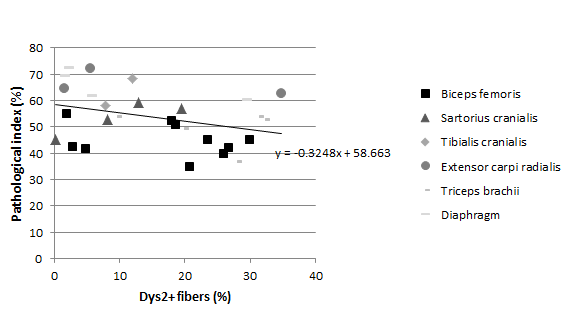

Supplement: Supplementary file 5 — Additional file 5: Figure S5. Correlation between Dp71 expression and histological lesions In 28 biopsies from 6 muscles sampled from 8 different LRMD dogs the proportion of Dys2+ fibres was quantified and compared to the pathological index on H&E stained serial sections. The correlation was not significant (Pearson’s R= -0.32; p =0.069). [file 13395_2020_239_MOESM5_ESM.tif]
