# Supplementary material for: X-linked muscular dystrophy in a Labrador Retriever strain: phenotypic and molecular characterisation
Source: Skelet Muscle. 2020 Aug 7;10:23. doi: 10.1186/s13395-020-00239-0 (PMC7412789; doi:10.1186/s13395-020-00239-0)
Supplement: Supplementary file 6 — Additional file 6. Table S1 [file 13395_2020_239_MOESM6_ESM.pdf]

Table S1 : sequences of PCR primers.

Use	Name of the primer	Sequence (5'-3')
Screening of the dystrophin cDNA by nested RT-PCRs<br><br>(Ref seq: ESNCAFT00000036277)	Ex3Fo	GGAAGCAGCACATAGAGAGAAC
	Ex3Fi	GAGACGCCTCCTAGACCTT
	Ex10Ro	TCACTTCTTCGACATCATTAG
	Ex10Ri	CTTCCAAAGCTGTTTGATAAC
	Ex10Fo	GTTATCAAACAGCTTTGGAAG
	Ex10Fi	CTAATGATGTCGAAGAAGTGA
	Ex15Fo	TTTCGGAGAAGGAAGATGCA
	Ex15Fi	GGCTTTAAGGATCAAAGTGA
	Ex20Ro	GATGATGTTGTTCTGATACTC
	Ex20Ri	CTTAGCAATTGGCAGAACTC
	Ex22Ro	CTCTGTTCCATGATGTCATATTC
	Ex22Ri	TGTCAGGATGGTACTCATAG
	Ex21Fo	CCTGAAAGAGAAAGGACAAG
	Ex21Fi	CTTTGTGGCCTTTACAAATC
	Ex26Ro	TCAACTGCTGTCTGTAATTC
	Ex26Ri	TCTTCAGCTTGTGTCATCCA
	Ex25Fo	TAGTCTCAACAGTGTCAATG
	Ex25Fi	CAGAAGATGAAGAATGAAGC
	Ex36Ro	CCAGTTACTATTCAGAAGAC
	Ex36Ri	CCACCAACATTTCCTTCTTG
	Ex35Fo	GCTTGAAATTGTCCCGTAAG
	Ex35Fi	CAGATATGGAACTGACAAAG
	Ex46Ro	GCATCTGTTTTTGAGGACTG
	Ex46Ri	GTTGCATTCAATACCCTGAC
	Ex45Fo	CAGAAGCTGAACAGTTTCTC
	Ex45Fi	CACAAATTCCTGAGAATTGG
	Ex56Ro	CTAGAAGCCTTTCCTTATGG
	Ex56Ri	GTTGTTTCGGCTTCTGTAAG
	Ex55Fo	GTGGCAGATAAATGTAGATG
	Ex55Fi	CTTCTCCGAGATTTATTCTGC
	Ex67Ro	GTACTTGTCTTCCAAATGGG
	Ex67Ri	GTTTTAAAAGACAGGACCCG
	Ex66Fo	CAACCTCAAGCAAAATGACC
	Ex66Fi	CTGTCTGACCACTATTTATG
	Ex79Ro	CTGAAACTAAGGACTCCATC
	Ex79Ri	GGAAAAGGCTTCCTACATTG
Screening of the intron 20 by PCR (Ref seq: ENSCAFG00000023562)	F1 (in Exon 20)	AAACTGGTTGAAAACCCAGC
	R1	TTATCACCCCTCTACCACCA
	F2	TGTCAAGGAACACGAGA
	R2	AGAAAAAGAGTAAGGGGTCAT
	F3	GAGGGGTTGAGATAAGATGA
	R3	GGAGGGGAAAATGGAAAGAA
	F4	ACAAGTGACAGTGCCCGTTA
	R4 (in Exon 21)	GCCCTTGTCCTTTCTCTTTC
Screening of the intron 20 by PCR (delineation of the mutation site)	F4i	GAGCAAACTCTCACTCTAAC
	R4i	CACATTACTTCTCCTGACTG
Distant breakpoint characterization by PCR (Ref seq: Ensembl CanFam3.1)	MutF	CAGATGTTAGGCCTTGGTTA
	MutR	GCATGAATGATGATTGCAATG
Dp71 RT-PCR (Ref seq: AY566609)	Dp71F	CAGCTCAAAGGCCACGAGAC
	Ex64-65R	AGGCTCAAGAGATCCAAGCA
