# Supplementary material for: X-linked muscular dystrophy in a Labrador Retriever strain: phenotypic and molecular characterisation
Source: Skelet Muscle. 2020 Aug 7;10:23. doi: 10.1186/s13395-020-00239-0 (PMC7412789; doi:10.1186/s13395-020-00239-0)
Supplement: Supplementary file 7 — Additional file 7. Table S2 [file 13395_2020_239_MOESM7_ESM.pdf]

Dogs with clinical, histopathological, immunohistochemical suspicion of DMD without mutation charcaterization

Breed	Clinical description	Histopathological description	Dystrophin absence IHC and/or WB	Specific observations	Reference
Irish terrier	x	x			[9]
WH Fox terrier	x	x	x		[11]
Miniature schnauzer	x	x	x		[12]
Samoyed	x	x			[13]
Gronendael	x	x			[14]
Brittany spaniel	x	x			[16]
Rat terrier	x	x	x		[18]
Labrador retriever	x	x	x		[19]
Old english shepherd dog	x	x	x		[20]
Weimaraner	x	x	x		[21]
Grand basset griffon vendéen	x	x	x		[22]
Alaskan Malamute	x	x	x		[25]
Labrador retriever	x	x	x	BMD-like IHC, WB: 135 kDa dystrophin	[26]
Labrador retriever	x	x	x	Very mild phenotype	[27]
Lurcher	x	x	x		[29]
Border Terrier	x	x	x	BMD-like	[35]

Dogs with confirmed and characterized DMD mutations

Breed	Mutation type	Location	Colony availability (to our knowledge)	phenotypic/functional description	Specific observations	Reference
Golden retriever 'GRMD'	Splice site point mutation	Intron 6	x	x	reference canine preclinical model for DMD	[5] [38]
Rottweiler	Non-sense mutation	Exon 58				[15]
German short-haired pointer	5.6 Mb deletion	Whole DMD gene + TMEM47 gene	x		no revertant fibers, potential interest immunological/ cognition?	[17] [36]
Welsh Corgi	Insertion (LINE-1 element)	Intron 13	x			[23]
Cavalier King Charles spaniel	Splice site mutation	Intron 50	x	ongoing	amenable to exon 51 skipping	[24]
Labrador retriever	184 bp insertion	Intron 19				[6] [37]
Cocker spaniel	4 bp deletion	Exon 65				[6]
Tibetan terrier	Deletion	Exons 8 to 29				[6]
Norfolk terrier	1 bp deletion	Exon 22				[28]
Japanese spitz	5.4 Mb inversion	Intron 19 -RPGR gene			Dp71 expression in skeletal muscles	[30]
Cavalier King Charles spaniel	7 bp deletion	Exon 42				[31]
Miniature Poodle	> 5Mb deletion	Whole DMD gene				[32]
Border Collie	1 bp deletion	Exon 20	semen archived			[33]
Labradoodle	Point mutation	Exon 21	x	x		[34]
Labrador retriever	*2.2 Mb inversion*	*Intron 20*	*x*	*x*	*Dp71 expression in skeletal muscles*	*present study*
